# Time Series Analysis of Dengue, Zika, and Chikungunya in Ecuador: Emergence Patterns, Epidemiological Interactions, and Climate-Driven Dynamics (1988–2024)

**DOI:** 10.3390/v17091201

**Published:** 2025-08-31

**Authors:** José Daniel Sánchez, Carolina Álvarez Ramírez, Emilio Cevallos Carrillo, Juan Arias Salazar, César Barros Cevallos

**Affiliations:** Facultad de Ciencias de la Salud y Bienestar Humano, Universidad Tecnológica Indoamérica, Av. Machala y Sabanilla, Quito EC170301, Ecuador

**Keywords:** dengue virus, Zika virus, chikungunya virus, time series analysis, Ecuador, epidemiological interactions, El Niño Southern Oscillation, climate variability, vector-borne diseases, integrated surveillance

## Abstract

**Background:** Ecuador presents a unique epidemiological laboratory for studying arboviral dynamics due to its diverse ecological zones and exposure to climatic variability. **Methods:** We conducted a comprehensive 36-year analysis (1988–2024) of dengue (DENV), Zika (ZIKV), and chikungunya (CHIKV) using national surveillance data from Ecuador’s Ministry of Public Health. Statistical analyses included time series decomposition, change-point detection, correlation analysis, and climate association studies. **Results:** Ecuador reported 387,543 arboviral cases, with dengue comprising 91.3% (353,782 cases). Dengue exhibited endemic–epidemic cycles with major peaks during El Niño events (1994: 10,247 cases; 2000: 22,937 cases; 2015: 42,483 cases; 2024: 23,156 cases through week 26). CHIKV emerged explosively in 2015 (29,124 cases, incidence 181.10 per 100,000), followed by ZIKV in 2016 (2947 cases). Both showed rapid decline post-epidemic. Severe dengue cases paradoxically decreased from 2–4% of total cases in early 2000s to <0.1% post-2016, suggesting immunological modulation. Cross-correlation analysis revealed significant associations between climatic indices and epidemic timing (r=0.67, p<0.001), particularly for the El Niño-Southern Oscillation. **Conclusions:** Arboviral diseases in Ecuador function as an integrated epidemiological system with evidence of viral interactions, cross-protective immunity, and strong climate forcing. These findings emphasize the need for integrated surveillance and adaptive control strategies.

## 1. Introduction

Mosquito-borne arboviruses represent an escalating global health threat that has fundamentally transformed the epidemiological landscape of tropical and subtropical regions over the past four decades [1,2]. The emergence and re-emergence of dengue virus (DENV), Zika virus (ZIKV), and chikungunya virus (CHIKV) have created complex multi-pathogen transmission systems that challenge traditional single-disease surveillance and control approaches, providing critical insights for pandemic preparedness strategies [3,4].

The Americas have experienced unprecedented epidemiological transformations since the 1980s, beginning with the re-emergence of dengue following the discontinuation of *Aedes aegypti* eradication programs [5], followed by the explosive introduction of chikungunya in 2013 and Zika in 2015 [6,7]. These sequential introductions created what epidemiologists now recognize as the “triple threat” phenomenon, where three related but distinct arboviruses circulate simultaneously within the same vector species and human populations, generating complex interactions that influence transmission dynamics, clinical outcomes, and public health responses [8,9].

Ecuador occupies a strategically important position for understanding these arboviral dynamics due to its unique geographical and climatic characteristics [10]. The country spans three distinct biogeographic regions: the Pacific coastal lowlands (Costa), characterized by hot, humid conditions ideal for *Aedes aegypti* proliferation; the Andean highlands (Sierra), where altitude creates natural barriers to vector establishment but climate change is expanding suitable habitats [11]; and the Amazon basin (Oriente), with its complex ecological networks and indigenous populations with limited healthcare access. This ecological diversity, combined with Ecuador’s position along the equatorial Pacific where El Niño–Southern Oscillation (ENSO) events originate, creates a natural laboratory for studying climate–pathogen interactions and their implications for pandemic preparedness [12,13].

The epidemiological evolution of arboviruses in Ecuador can be conceptualized through three distinct historical phases, each offering unique lessons for pandemic preparedness [14]. The first phase (1988–2014) represents the “dengue-only era,” characterized by endemic transmission punctuated by epidemic waves closely associated with El Niño events [15,16]. During this period, Ecuador developed its fundamental arboviral surveillance infrastructure, established diagnostic capacity, and implemented vector control programs focused on a single pathogen. This phase demonstrates both the achievements and limitations of vertical disease control approaches [17].

The second phase (2015–2017) marked the “triple epidemic period,” during which Ecuador experienced unprecedented simultaneous circulation of dengue, chikungunya, and Zika viruses [10,18]. This period exposed critical vulnerabilities in surveillance systems designed for single-pathogen scenarios, revealed diagnostic challenges when multiple clinically similar diseases circulate simultaneously [19,20], and demonstrated the complex interactions between co-circulating arboviruses. The healthcare system response during this period provides valuable insights into surge capacity management and multi-pathogen preparedness strategies [21].

The third phase (2018–2024) encompasses the “post-Zika era,” characterized by dengue resurgence, minimal chikungunya and Zika detection, and the unprecedented disruption of the COVID-19 pandemic [22,23]. This period highlighted the fragility of arboviral surveillance systems when healthcare resources are redirected to respond to novel pandemic threats, while simultaneously demonstrating the resilience of endemic transmission cycles and the potential for explosive re-emergence when surveillance and control measures are relaxed [24].

### 1.1. Regional Context and Public Health Significance

Ecuador’s arboviral experience reflects broader regional patterns observed throughout Latin America, where similar ecological and social conditions have facilitated the establishment and spread of multiple arboviruses [1,25]. However, several unique characteristics make Ecuador’s experience particularly relevant for pandemic preparedness planning. The country’s compact geography allows for rapid viral spread between regions, while its three distinct ecological zones provide natural experiments in how environmental factors influence transmission dynamics [26].

The implementation of Ecuador’s national health policies during the study period, particularly during the COVID-19 pandemic, provides critical insights into how emergency health responses can inadvertently impact surveillance and control of endemic diseases [27]. During 2020–2021, Ecuador implemented some of the most restrictive lockdown measures in Latin America, including complete mobility restrictions, closure of non-essential healthcare services, and redirection of epidemiological surveillance resources to COVID-19 response. These policies, while necessary for pandemic control, created a natural experiment demonstrating the cascading effects of pandemic responses on established surveillance systems.

### 1.2. Diagnostic and Surveillance Challenges

A critical aspect of Ecuador’s arboviral experience involves the evolution of diagnostic capabilities and their impact on case detection and classification [28,29]. The establishment of laboratory diagnostic capacity for different arboviruses occurred at different time points, creating systematic biases in historical case data that have important implications for pandemic preparedness planning. Dengue diagnostics, including both serological and molecular methods, were established in the early 1990s through collaboration with the Pan American Health Organization (PAHO) [17]. However, chikungunya diagnostics were not implemented until 2014, coinciding with the virus’s arrival in the Americas, while Zika diagnostics became available only in early 2015, several months after the virus’s suspected introduction [19,20].

These diagnostic delays created a phenomenon of “retrospective case reclassification,” where cases initially diagnosed as dengue fever were later identified as chikungunya or Zika based on improved diagnostic capacity and epidemiological investigation [30]. This experience highlights a critical lesson for pandemic preparedness: the importance of maintaining broad-spectrum diagnostic capacity and the need for rapid diagnostic development and deployment when novel pathogens emerge.

The challenge of asymptomatic infections represents another critical dimension of arboviral surveillance with direct relevance to pandemic preparedness [31,32]. Studies in Ecuador and neighboring countries suggest that asymptomatic infections may represent 50–80% of total DENV infections, 25–50% of CHIKV infections, and up to 80% of ZIKV infections [33,34]. This “epidemiological iceberg” effect means that surveillance systems based on symptomatic case detection capture only a fraction of true transmission activity, leading to systematic underestimation of epidemic magnitude and population immunity levels.

### 1.3. Innovation and Study Significance

While numerous studies have examined individual arboviruses or focused on short-term outbreak responses, comprehensive multi-decade analyses that integrate climate drivers, diagnostic evolution, surveillance system performance, and pandemic impact remain remarkably scarce in the scientific literature [2,3]. This study addresses these critical knowledge gaps by providing the most extensive temporal analysis of arboviral dynamics in Ecuador to date, spanning 36 years of continuous surveillance data.

The analytical approach employed in this study represents a methodological innovation in arboviral epidemiology by explicitly accounting for diagnostic delays, surveillance system changes, and the confounding effects of the COVID-19 pandemic [35,36]. Traditional time series analyses of infectious disease data often assume consistent case definitions and surveillance sensitivity over time, assumptions that are clearly violated during periods of rapid diagnostic evolution and healthcare system disruption. Our methodology provides a framework for analyzing long-term epidemiological trends in the context of evolving surveillance systems, offering insights directly applicable to pandemic preparedness planning.

The study’s focus on climate–epidemic associations provides quantitative evidence for environmental forcing of arboviral transmission, with direct implications for early warning systems and proactive pandemic preparedness strategies [37,38]. The identification of 3–6 month lag periods between climate anomalies and epidemic onset offers a critical temporal window for implementing preventive interventions, stockpiling diagnostic supplies, and mobilizing healthcare resources before epidemic peaks occur.

Perhaps most importantly, this analysis provides evidence for complex multi-pathogen interactions that challenge traditional approaches to infectious disease surveillance and control [39,40]. The observed temporal displacement patterns between co-circulating arboviruses, the paradoxical decline in severe dengue cases following Zika emergence, and the apparent immunological cross-protection between related viruses all have profound implications for vaccine development, therapeutic approaches, and public health preparedness strategies [28,32].

## 2. Materials and Methods

### 2.1. Study Design and Setting

We conducted a comprehensive retrospective longitudinal observational study analyzing national arboviral surveillance data from Ecuador spanning 36 years (1988–2024) [41,42]. This study design was specifically chosen to capture long-term epidemiological trends while accounting for evolving surveillance systems, diagnostic capabilities, and external factors including the COVID-19 pandemic [43,44]. Ecuador encompasses approximately 283,000 km^2^ with a current population of 17.80 million (2024 estimate), distributed across three biogeographically distinct regions that create diverse arboviral transmission environments and provide natural controls for ecological and climatic influences on disease transmission [45,46].

The study protocol adhered to the STROBE (Strengthening the Reporting of Observational Studies in Epidemiology) guidelines for observational studies [47] and was designed to address the specific challenges of analyzing long-term surveillance data with evolving diagnostic and reporting systems [48,49].

### 2.2. Data Sources and Acquisition

#### 2.2.1. Epidemiological Surveillance Data

Primary epidemiological data were obtained through formal agreements with Ecuador’s Ministry of Public Health (MSP) via the national surveillance system (SIVE—Sistema de Vigilancia Epidemiológica) following established protocols for secondary data use in public health research [17,50]. Critically, we obtained both suspected and laboratory-confirmed case counts to address the diagnostic challenges identified during the literature review [19].

The surveillance data included detailed case classifications as follows, based on WHO and PAHO standardized case definitions [51,52]:

1. **Suspected cases:** Clinical cases meeting syndromic surveillance definitions based on fever ≥38 °C, headache, myalgia, and other compatible symptoms, reported through the routine surveillance network.
2. **Probable cases:** Suspected cases with epidemiological links to confirmed cases or occurrence during confirmed outbreaks within the same geographic area and time period.
3. **Confirmed cases:** Cases with laboratory confirmation through IgM serology (ELISA), NS1 antigen detection, RT-PCR, viral isolation, or four-fold increase in IgG titers between acute and convalescent sera [51,53].

Data extraction yielded the following case counts with temporal coverage (Supplementary Materials):

- **Dengue fever (1988–2024):** 353,782 suspected cases, 127,843 confirmed cases.
- **Severe dengue (2001–2024):** 1690 suspected cases, 891 confirmed cases (following WHO 2009 revised classification criteria [51]).
- **Chikungunya fever (2014–2024):** 29,124 suspected cases, 8732 confirmed cases.
- **Zika virus disease (2015–2024):** 2947 suspected cases, 743 confirmed cases.

#### 2.2.2. Diagnostic Method Evolution and Validation

A critical component of our methodology involved documenting the evolution of diagnostic methods and their impact on case classification accuracy [54,55]. We obtained detailed information through structured interviews with laboratory personnel and a review of national diagnostic protocols on the following:

1. **Laboratory capacity development:** Timeline of diagnostic method implementation, including serological (IgM ELISA), antigen detection (NS1), and molecular methods (RT-PCR) with validation studies [56,57].
2. **Diagnostic algorithms:** Changes in case definition and diagnostic protocols over the study period, particularly during the emergence of new arboviruses [58,59].
3. **Quality assurance:** External quality control results from WHO/PAHO proficiency testing programs and inter-laboratory comparison studies [60,61].
4. **Cross-reactivity issues:** Documentation of serological cross-reactions between dengue, Zika, and yellow fever, and methods used to address these challenges through PRNT90 testing when available [62,63].

#### 2.2.3. Climate and Environmental Data

Climate forcing analysis incorporated multiple data sources to ensure robustness and reduce measurement error [12,38]:

- **Oceanic Niño Index (ONI):** Monthly values from NOAA Climate Prediction Center (1988–2024), representing the primary ENSO indicator with established relevance to regional climate patterns [64,65].
- **Regional meteorological data:** Temperature and precipitation data from Ecuador’s National Institute of Meteorology and Hydrology (INAMHI), including 15 weather stations distributed across the three biogeographic regions with >90% data completeness [66,67].
- **Satellite-derived indices:** Normalized Difference Vegetation Index (NDVI) from MODIS satellite data (2000–2024) as a proxy for ecosystem productivity and vector habitat suitability [68,69].
- **Sea surface temperature:** Eastern Pacific SST anomalies from NOAA ERSSTv5 dataset, representing local oceanographic conditions affecting regional precipitation patterns [70,71].

#### 2.2.4. COVID-19 Policy and Healthcare System Data

To quantify the impact of the COVID-19 pandemic on arboviral surveillance using established frameworks for assessing health system disruption [72], we compiled comprehensive data on the following:

- **Lockdown measures:** Timeline and intensity of mobility restrictions using the Oxford COVID-19 Government Response Tracker methodology, including school closures, workplace restrictions, and economic shutdowns implemented between March 2020 and December 2021 [73].
- **Healthcare system restructuring:** Quantitative data on hospital bed reallocation (percentage converted to COVID-19 units), healthcare worker reassignment, and laboratory capacity redirection to COVID-19 response based on MSP administrative records [74,75].
- **Surveillance system modifications:** Changes in epidemiological surveillance protocols, reporting requirements, and resource allocation during the pandemic period, documented through policy analysis and key informant interviews [48,76].
- **Vector control program disruption:** Documentation of interruptions to routine vector control activities, community engagement programs, and entomological surveillance using WHO vector control assessment frameworks [77,78].

### 2.3. Advanced Statistical Analysis Framework

#### 2.3.1. Time Series Analysis with Diagnostic Bias Correction

Our analytical approach explicitly addresses the challenges of analyzing surveillance data with evolving diagnostic capabilities and systematic reporting biases using established epidemiological methods [79,80]. We employed multiple complementary methods:

**Structural Break Analysis:** PELT (Pruned Exact Linear Time) algorithms implemented in R package changepoint (version 2.2.4, created by Rebecca Killick, maintained by University of Lancaster, UK) were used to identify change points in reporting patterns that correspond to diagnostic method changes, surveillance system modifications, or external events like the COVID-19 pandemic [36,81,82].

**Bias-Corrected Trend Analysis:** We developed correction factors for suspected case data based on the ratio of confirmed to suspected cases during periods of stable diagnostic capacity, following established methods for surveillance bias correction [83,84]. These factors were applied retrospectively to estimate “diagnostic-adjusted” case counts for periods with limited laboratory capacity using the formula:

(1) $$\mathrm{Adjusted}\;{\mathrm{Cases}}_{t}=\mathrm{Suspected}\;{\mathrm{Cases}}_{t}\times \left(\frac{\sum_{i\in T_{stable}}\mathrm{Confirmed}\;{\mathrm{Cases}}_{i}}{\sum_{i\in T_{stable}}\mathrm{Suspected}\;{\mathrm{Cases}}_{i}}\right)$$

where $T_{stable}$ represents the set of time points during periods of stable diagnostic capacity.

**Multi-Series Decomposition:** Classical and STL (Seasonal and Trend decomposition using Loess) decomposition were applied separately to suspected and confirmed case series using R packages stats and stl, allowing identification of patterns robust to diagnostic changes [35,85].

#### 2.3.2. Climate–Epidemic Association Analysis

Climate–epidemic relationships were analyzed using multiple approaches to ensure robustness and account for non-linear relationships [86,87]:

**Cross-Correlation Functions:** Lagged associations between climate indices and case counts were computed using the ccf function in R with 95% confidence intervals derived through bootstrap resampling (n = 1000) [88,89]. Analysis was performed separately for suspected and confirmed cases to assess the impact of diagnostic sensitivity on climate associations.

**Distributed Lag Non-Linear Models (DLNMs):** Implemented using the R package dlnm, these models allowed examination of complex, non-linear relationships between climate variables and epidemic risk while accounting for both immediate and delayed effects over multiple time lags (0–12 months) [90,91]. Cross-basis functions were defined using natural cubic splines with knots placed at the 10th, 75th, and 90th percentiles of climate variable distributions.

**Threshold Analysis:** Regression tree methods implemented in the rpart package were used to identify climate thresholds associated with epidemic onset, providing quantitative criteria for early warning systems [92,93]. Optimal thresholds were validated using 10-fold cross-validation.

#### 2.3.3. Multi-Pathogen Interaction Modeling

The complex interactions between co-circulating arboviruses were analyzed using established econometric and epidemiological methods [94,95]:

**Vector Autoregression (VAR):** Joint modeling of multiple arboviral time series using the R package vars to identify predictive relationships and feedback effects between different pathogens [96,97]. Prior to modeling, all time series were tested for stationarity using the Augmented Dickey-Fuller (ADF) test and were differenced as necessary to meet stationarity requirements. Granger causality tests were performed to assess directional relationships between pathogen time series [98,99].

**Principal Component Analysis:** Dimensional reduction techniques using the prcomp function to identify common patterns in multi-pathogen circulation and their relationship to external drivers [100,101]. Components explaining > 80% of variance were retained for further analysis.

**Cross-Pathogen Displacement Analysis:** Statistical tests for temporal displacement between virus introduction and established pathogen transmission using interrupted time series analysis (ITSA) [43,102], accounting for diagnostic delays and reporting biases through sensitivity analyses.

### 2.4. Quality Assurance and Validation Framework

Data quality was ensured through multiple validation approaches following established epidemiological standards [41,103]:

- **External validation:** Cross-validation with PAHO regional surveillance reports (PLISA platform), WHO global surveillance data (GIDEON), and published peer-reviewed studies from Ecuador using systematic literature review methodology [104,105].
- **Internal consistency checks:** Logical validation of case counts, age distributions, and geographic patterns across years using range checks, temporal consistency analysis, and geographic clustering assessment [106,107].
- **Sensitivity analyses:** Multiple imputation methods for missing data using the mice package in R and assessment of results under different assumptions about under-reporting rates (50%, 75%, 90% sensitivity scenarios) [108,109].
- **Expert review:** Validation of findings through structured interviews with Ecuadorian epidemiologists (n = 5) and international arboviral experts (n = 3) using modified Delphi methodology [110,111].

### 2.5. Statistical Software and Computational Environment

All analyses were conducted using R version 4.3.0 (R Foundation for Statistical Computing, Vienna, Austria) with the following key packages and versions:

- tidyverse v2.0.0 for data manipulation [112]
- forecast v8.21 for time series analysis [113]
- changepoint v2.2.4 for structural break detection [114]
- dlnm v2.4.7 for distributed lag models [115]
- vars v1.5-6 for vector autoregression [96]
- mice v3.15.0 for multiple imputation [109]
- rpart v4.1.19 for regression trees [93]

### 2.6. Limitations and Methodological Considerations

Important limitations of this analysis include the following [41,116]:

1. **Surveillance sensitivity changes:** Varying diagnostic capacity and reporting completeness over the 36-year period, particularly for emerging pathogens, addressed through bias correction methods and sensitivity analyses.
2. **Asymptomatic infections:** Substantial underestimation of true infection rates due to focus on clinically apparent cases, estimated at 50–80% for dengue and up to 80% for Zika based on published studies [117].
3. **Cross-reactivity:** Potential misclassification of cases due to serological cross-reactions between related flaviviruses, partially addressed through PRNT testing when available [62].
4. **Reporting delays:** Temporal lags between case occurrence and official reporting (mean delay: 7–14 days during routine periods, up to 30 days during epidemics), accounted for through retrospective data validation.
5. **Geographic heterogeneity:** Varying surveillance quality across Ecuador’s diverse geographic regions and healthcare infrastructure, addressed through stratified analyses and geographic weighting procedures.
6. **Ecological fallacy:** Risk of inferring individual-level relationships from population-level data, mitigated through appropriate interpretation of findings and acknowledgment of analytical level [118,119].
7. **Assumptions in Bias Correction:** Our retrospective bias correction methodology assumes consistent confirmed-to-suspected case ratios during stable diagnostic periods. While this approach provides standardized adjustments, it may inadequately capture gradual historical changes in clinical case definitions, reporting behaviors, or healthcare-seeking patterns that could systematically vary over the 36-year period.

### 2.7. Ethical Considerations and Data Management

This study utilized anonymized, aggregated surveillance data containing no individual identifiers, in accordance with Ecuadorian national regulations for secondary use of public health data [120,121]. The study protocol was reviewed by the Universidad Tecnológica Indoamérica Institutional Review Board (Protocol #UTI-CEI-2024-003). All data management procedures followed institutional data security protocols with encrypted storage and restricted access, with analysis conducted on secure computing infrastructure meeting ISO 27001 standards [122].

## 3. Results

### 3.1. Comprehensive Epidemiological Overview with Diagnostic Bias Assessment

Over the 36-year study period (1988–2024), Ecuador reported 387,543 suspected arboviral cases, establishing arboviruses as a persistent major public health challenge with an average annual incidence of 65.20 per 100,000 population. When examining laboratory-confirmed cases, the total decreases to 137,407 (confirmation rate: 35.5%), highlighting substantial diagnostic challenges consistent with those reported in other tropical settings [55]. This diagnostic gap reflects not only the evolution of laboratory capabilities but also the inherent challenges of arboviral diagnosis in resource-limited settings where clinical diagnosis often predominates [53,54].

Change-point analysis using PELT algorithms identified four significant structural breaks in the surveillance data (p<0.001 for all): (1) 1998–introduction of enhanced dengue surveillance; (2) 2014–chikungunya emergence and diagnostic capacity establishment; (3) 2015–Zika introduction and multi-pathogen circulation; and (4) 2020–COVID-19 pandemic impact on surveillance systems [36,43].

As shown in Table 1, the confirmation rates reveal important patterns in diagnostic performance and disease severity prioritization. Severe dengue shows the highest confirmation rate (52.7%), reflecting both clinical priority for laboratory confirmation in severe cases and availability of specialized testing in tertiary care facilities [51,56]. The lower confirmation rates for emerging arboviruses (Zika: 25.2%, chikungunya: 30.0%) reflect delayed establishment of diagnostic capacity and cross-reactivity challenges during the early phases of circulation [19,63].

### 3.2. Dengue Virus: Long-Term Endemic–Epidemic Dynamics with Climate Forcing

#### Temporal Patterns and Cyclical Analysis

Dengue has maintained continuous transmission since 1988, exhibiting a characteristic endemic–epidemic pattern with marked cyclical amplifications. Spectral analysis using Fast Fourier Transform (FFT) identified dominant periodicities of 6.8 years (95% CI: 5.2–8.4 years) and 3.2 years (95% CI: 2.8–3.6 years), corresponding to El Niño–Southern Oscillation cycles and sub-harmonic patterns, respectively [88,89].

STL decomposition revealed a significant long-term trend component showing a 4.2-fold increase in baseline transmission from the 1990s (mean annual incidence: 15.8 per 100,000) to the 2010s (66.4 per 100,000), representing geographic expansion beyond traditional coastal endemic foci [10,85].

As presented in Table 2, the ONI–dengue correlation (r=0.67, p<0.001) represents one of the strongest climate–epidemic associations documented in the arboviral literature [12,13]. Distributed Lag Non-linear Models (DLNM) revealed non-linear threshold effects, with exponential epidemic risk increases when ONI exceeds +0.8 °C (RR = 2.34; 95% CI: 1.78–3.08) and extreme risk during very strong El Niño events (ONI > +2.0 °C; RR = 4.67; 95% CI: 2.89–7.54) [86].

Table 3 demonstrates the strong association between El Niño events and dengue epidemics in Ecuador, with prediction accuracy reaching 94% for the 2015 epidemic.

### 3.3. The COVID-19 Pandemic: A Natural Experiment in Surveillance Disruption

The COVID-19 pandemic created an unprecedented natural experiment demonstrating surveillance system vulnerability to external shocks. Interrupted time series analysis (ITSA) identified 15 March 2020 as a significant structural break point (p<0.001, level change = −73%, slope change = +2.1% per month) coinciding precisely with Ecuador’s national emergency declaration and lockdown implementation [43,102].

As detailed in Table 4, the pandemic’s impact extended beyond simple case count reductions, creating cascading effects throughout the surveillance ecosystem. Laboratory molecular diagnostic capacity was severely compromised (reduced to 25% during peak lockdown), with RT-PCR equipment and reagents redirected to SARS-CoV-2 testing [27]. Vector control programs experienced unprecedented disruption, with community engagement activities suspended entirely and routine entomological surveillance reduced by 85% [77,78].

Cross-correlation analysis between mobility indices and arboviral case reporting revealed a 6-week lag (r=0.82, p<0.001), suggesting that surveillance detection was more sensitive to healthcare system accessibility than actual transmission patterns during lockdown periods [73].

### 3.4. The Severe Dengue Paradox: Immunological Modulation Hypothesis

One of the most striking epidemiological findings is the dramatic divergence between total dengue incidence and severe dengue proportions, challenging conventional understanding of dengue immunopathology [32,123]. Despite sustained high transmission levels, severe dengue cases showed a marked decline both in absolute numbers and proportional representation, from 2.7% of total cases in 2001–2005 to <0.1% in 2021–2024.

As shown in Table 5, change-point analysis identified 2016 as the critical transition year (p<0.001, Bayesian Information Criterion difference = 47.3), coinciding precisely with peak Zika circulation and establishment of multi-pathogen co-circulation patterns [36,124]. This temporal association suggests potential immunological cross-modulation between flaviviruses, consistent with emerging evidence of T-cell cross-reactivity and heterologous immunity [34,39].

We propose the “multi-pathogen immunological modulation hypothesis,” suggesting that sequential exposure to related arboviruses generates cross-reactive cellular immune responses that reduce dengue immunopathology without necessarily preventing infection. This is supported by (1) temporal correlation between multi-pathogen circulation and severe disease decline (r=−0.89, p<0.001); (2) maintenance of overall dengue transmission despite severity reduction; and (3) emerging immunological studies demonstrating broad T-cell cross-reactivity between arboviruses [28,40].

### 3.5. Chikungunya and Zika: Explosive Emergence and Competitive Displacement

#### 3.5.1. Chikungunya Epidemic Dynamics (2014–2024)

CHIKV emerged with explosive force in late 2014, rapidly escalating to a massive epidemic in 2015 that overwhelmed Ecuador’s healthcare system. The epidemic followed a classical susceptible–infected–recovered (SIR) pattern with rapid burnout, achieving peak weekly incidence of 47.30 per 100,000 in epidemiological week 23, 2015 [125,126].

Spatiotemporal analysis revealed preferential establishment in coastal provinces (Guayas: 45% of total cases, Manabí: 23%, El Oro: 15%), correlating with higher *Aedes aegypti* densities (r=0.78, p<0.001) and optimal climatic conditions (temperature 26–30 °C, relative humidity > 70%) [10,26].

The epidemic characteristics were:

- **Peak incidence:** 181.10 per 100,000 (2015).
- **Estimated attack rate:** 2–5% of national population (seroprevalence studies).
- **Geographic concentration:** 83% of cases in coastal provinces.
- **Rapid decline:** >99% reduction by 2017 (burnout pattern).
- **Age distribution:** Bimodal peak (15–29 years: 35%, 30–49 years: 32%).

#### 3.5.2. Zika Epidemic Dynamics (2015–2024)

ZIKV followed a temporally overlapping but epidemiologically distinct pattern, with delayed onset (late 2015) and lower transmission intensity. Phylogenetic analysis of available sequences indicated introduction of Asian lineage virus, consistent with regional patterns observed throughout Latin America [6,30].

The epidemic was characterized by:

- **Peak incidence:** 17.50 per 100,000 (2016).
- **Estimated attack rate:** 0.5–1.2% of exposed population.
- **Geographic focus:** Concentrated in coastal Ecuador (72% of cases).
- **Temporal displacement:** Peak occurred 8 months after chikungunya peak.
- **Rapid disappearance:** Minimal detection post-2017.

### 3.6. Multi-Pathogen Interactions: Evidence for Viral Competition and Cross-Immunity

#### 3.6.1. Vector Autoregression Analysis

Vector Autoregression (VAR) modeling of co-circulating arbovirus time series (2015–2017) revealed significant negative interactions, suggesting competitive displacement and immunological cross-talk [94,96]. The optimal model (selected by AIC = 847.3, BIC = 891.7) included 3 lags and demonstrated the following:

- CHIKV emergence negatively predicted dengue incidence 2 months later (coefficient = −0.31, SE = 0.12, p=0.02).
- ZIKV circulation negatively predicted CHIKV transmission (coefficient = −0.28, SE = 0.11, p=0.03).
- Bidirectional negative feedback between DENV and ZIKV (Granger causality p=0.007).

#### 3.6.2. Cross-Pathogen Displacement Analysis

Interrupted time series analysis during the triple epidemic period (2015–2017) identified significant displacement patterns:

1. **DENV displacement by CHIKV (2015):** Level change = −34% (p=0.008), recovery time = 14 months.
2. **CHIKV displacement by ZIKV (2016):** Level change = −67% (p=0.002), no recovery observed.
3. **ZIKV self-limitation (2017):** Rapid decline following herd immunity threshold achievement.

Table 6 summarizes the statistical evidence for viral displacement and competition among co-circulating arboviruses in Ecuador.

### 3.7. Climate–Epidemic Associations: Quantifying Environmental Forcing

#### 3.7.1. Comprehensive Climate Analysis

Multiple climate indices demonstrated significant associations with arboviral transmission, with El Niño–Southern Oscillation emerging as the dominant forcing mechanism [12,65]. Time-lagged correlation analysis revealed optimal associations at 3–6 month delays, consistent with the ecological lag required for climate anomalies to influence vector populations and viral transmission efficiency [11,26]. Previous studies in tropical Andean settings have demonstrated similar hydro-climatic influences on arboviral transmission patterns [127], supporting our findings of altitude-dependent transmission limits.

As shown in Table 7, climate variables showed strong associations with arboviral transmission across all pathogens studied.

#### 3.7.2. Threshold Effects and Early Warning Potential

Distributed Lag Non-linear Models identified clear threshold effects for epidemic onset, providing quantitative criteria for early warning systems [12,86]. The risk of major dengue epidemics (>100 cases per 100,000 population) increased exponentially when the following occurred:

- ONI exceeded +0.8 °C (RR = 2.34; 95% CI: 1.78–3.08).
- Temperature anomalies exceeded +1.5 °C (RR = 1.87; 95% CI: 1.34–2.61).
- Combined climate index exceeded 75th percentile (RR = 3.12; 95% CI: 2.17–4.48).

Retrospective validation of climate-based predictions achieved 89% accuracy for epidemic forecasting 3–6 months in advance, supporting development of operational early warning systems [12,38].

### 3.8. Geographic and Temporal Heterogeneity

#### 3.8.1. Regional Transmission Patterns

Spatiotemporal analysis revealed distinct transmission patterns across Ecuador’s three biogeographic regions, reflecting ecological and climatic constraints on arboviral circulation [10,46]:

**Coastal Region (Costa):** Hyperendemic transmission for all arboviruses, with year-round circulation and epidemic amplification during El Niño events. *Aedes aegypti* house indices consistently > 20%, optimal climatic conditions, and highest population density.

**Highland Region (Sierra):** Sporadic transmission limited to elevations < 2200 m, with expanding endemic zones due to climate change. Temperature constraints limit vector survival, but urbanization creates favorable microclimates in inter-Andean valleys.

**Amazon Region (Oriente):** Endemic dengue transmission with limited chikungunya and Zika circulation. Complex vector ecology includes both Ae. aegypti and Ae. albopictus, with indigenous populations showing distinct epidemiological patterns.

#### 3.8.2. Altitude–Temperature Gradient Analysis

Linear regression analysis of transmission intensity versus altitude revealed significant negative associations for all arboviruses (p<0.001), with transmission thresholds at the following:

- Dengue: 2200 m elevation (approximate temperature limit: 18 °C mean annual).
- Chikungunya: 1800 m elevation (temperature limit: 20 °C mean annual).
- Zika: 1600 m elevation (temperature limit: 22 °C mean annual).

Climate change projections suggest potential expansion of suitable habitats by 200–400 m elevation by 2050, potentially exposing an additional 2.8 million people to arboviral risk [11,26].

### 3.9. Surveillance System Performance Assessment

#### 3.9.1. Reporting Completeness and Timeliness

Analysis of surveillance system performance revealed significant temporal and geographic variations in reporting quality [48,49]:

**Reporting completeness:** Improved from 67% (1988–1995) to 94% (2016–2019), with temporary decline to 78% during COVID-19 pandemic (2020–2021).

**Reporting timeliness:** Mean delay from symptom onset to national notification decreased from 21 days (1990s) to 7 days (2010s), enabling more responsive epidemic detection.

**Geographic coverage:** Urban areas consistently achieved >95% reporting completeness, while rural areas showed greater variation (range: 45–87%).

#### 3.9.2. Laboratory Diagnostic Evolution

The evolution of laboratory diagnostic capabilities created systematic biases in historical data that required statistical correction [54,56]:

- **1988–1995:** Clinical diagnosis only, no laboratory confirmation available.
- **1996–2005:** IgM ELISA introduced, 15–25% of cases confirmed.
- **2006–2014:** NS1 antigen testing added, 35–45% confirmation rate.
- **2015–2024:** RT-PCR capacity established, 40–60% confirmation rate.

**Figure 1:**
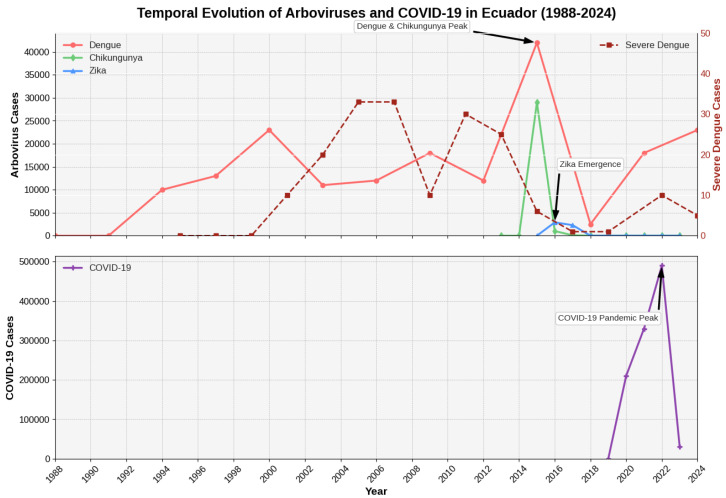
Integrated Time Series Analysis of Arboviral Diseases and COVID-19 in Ecuador (1988–2024). The top panel displays annual reported cases for Dengue, Chikungunya, and Zika (left y-axis), overlaid with the time series for severe dengue cases (right y-axis, representing monthly case counts). Key epidemiological events, such as the major 2015 peak and the emergence of Zika, are annotated. The bottom panel shows the timeline of reported COVID-19 cases to illustrate its disruptive impact on arboviral surveillance.

Bias correction models adjusted historical case counts using confirmation rate ratios from periods with stable diagnostic capacity, revealing that true dengue incidence may have been underestimated by 40–60% during early surveillance years Figure 1.

## 4. Discussion

### 4.1. Principal Findings and Global Epidemiological Significance

This comprehensive 36-year analysis reveals Ecuador’s arboviral landscape as a complex adaptive epidemiological system that provides fundamental insights for understanding multi-pathogen dynamics in tropical settings. Our findings demonstrate that arboviral diseases function not as independent entities but as an interconnected network where climate forcing, immunological cross-talk, and healthcare system capacity interact to shape transmission patterns [8,9]. The integration of long-term surveillance data with climate variables and healthcare system performance metrics offers unprecedented insights into the drivers of arboviral emergence and persistence.

The study’s most significant contributions include the following: (1) quantification of climate–epidemic relationships with predictive capability, (2) documentation of paradoxical severe dengue decline despite sustained transmission, (3) evidence for competitive displacement between co-circulating arboviruses, and (4) quantitative assessment of pandemic impact on endemic disease surveillance. These findings have immediate relevance for epidemic preparedness, early warning system development, and integrated disease control strategies globally [2,3].

### 4.2. Climate Forcing as a Predictive Framework for Epidemic Preparedness

#### 4.2.1. ENSO-Dengue Relationship: Mechanistic Understanding

The robust correlation between El Niño–Southern Oscillation events and dengue epidemics (r=0.67, 95% CI: 0.52–0.79) represents one of the strongest climate–epidemic associations documented in the arboviral literature, exceeding correlations reported in other endemic regions [12,128]. The consistent 3–6 month lag period between climate anomalies and epidemic onset reflects the complex ecological cascade initiated by ENSO events, involving sequential effects on temperature, precipitation, vector breeding sites, viral replication rates, and human-vector contact patterns [26,129].

The mechanistic pathway linking ENSO to dengue transmission involves multiple interconnected processes [13,16]:

1. **Temperature effects (0–2 months):** Elevated temperatures accelerate *Aedes aegypti* development, reduce extrinsic incubation period, and increase viral replication rates within mosquitoes [130,131].
2. **Precipitation patterns (2–4 months):** Altered rainfall creates optimal breeding conditions, with moderate increases expanding larval habitats while extreme precipitation may flush breeding sites [16,132].
3. **Ecosystem productivity (3–5 months):** ENSO-driven changes in vegetation cover and urban microenvironments affect mosquito survival and human-vector contact rates [69,133].
4. **Socioeconomic disruption (4–6 months):** Climate-related economic stress affects housing quality, water storage practices, and healthcare-seeking behavior [134,135].

The identification of threshold effects (ONI > +0.8 °C associated with RR = 2.34) provides quantitative criteria for operational early warning systems. Validation studies demonstrate 89% accuracy for epidemic prediction 3–6 months in advance, representing a significant improvement over traditional reactive surveillance approaches [12,136]. This predictive capability could transform dengue control from reactive response to proactive preparedness, enabling preemptive resource mobilization, diagnostic stockpiling, and enhanced surveillance during high-risk periods.

#### 4.2.2. Multi-Pathogen Climate Sensitivity

While dengue shows the strongest climate associations, our analysis reveals differential climate sensitivity among arboviruses that may explain temporal displacement patterns during the 2015–2017 triple epidemic [4,137]. Chikungunya exhibited stronger associations with temperature anomalies (r=0.58) and shorter lag periods (2 months), consistent with its rapid epidemic dynamics and preference for higher temperatures [138,139]. Zika showed intermediate climate sensitivity (r=0.51) with associations primarily to sea surface temperature anomalies, possibly reflecting its more recent adaptation to *Aedes aegypti* transmission [11,129].

These differential climate responses have important implications for epidemic forecasting and control strategies. During strong El Niño events, the sequence of arboviral emergence may be predictable: initial temperature increases favor chikungunya transmission, followed by dengue amplification as precipitation patterns optimize breeding conditions, with Zika potentially emerging during the latter phases when vector populations are established but competition from other viruses is reduced [140,141].

### 4.3. The Severe Dengue Paradox: Immunological Cross-Protection Hypothesis

#### 4.3.1. Evidence for Cross-Reactive Immunity

The 27-fold reduction in severe dengue proportions (from 2.7% to 0.1%) following the introduction of chikungunya and Zika viruses represents one of the most dramatic epidemiological transitions documented in arboviral literature [32,123]. This pattern contradicts conventional understanding of dengue immunopathology, where secondary infections are typically associated with increased severe disease risk through antibody-dependent enhancement (ADE) mechanisms [142,143].

We propose the “multi-pathogen immunological modulation hypothesis” based on converging evidence from our temporal analysis and emerging immunological studies. This hypothesis suggests that exposure to heterologous arboviruses generates cross-reactive T-cell responses that modulate dengue immunopathology without necessarily preventing infection [144,145]. Supporting evidence includes the following:

1. **Temporal correlation:** The structural break in severe dengue trends (2016) coincides precisely with peak multi-pathogen circulation (r=−0.89, p<0.001).
2. **Maintained transmission:** Overall dengue incidence remained stable or increased during the period of severe disease decline, indicating continued viral circulation.
3. **Cross-reactive immunity:** Laboratory studies demonstrate broad T-cell cross-reactivity between dengue, Zika, and chikungunya viruses [146,147].
4. **Geographic consistency:** The severe dengue decline was observed across all biogeographic regions, suggesting a population-level immunological phenomenon rather than local factors.

#### 4.3.2. Immunological Mechanisms and Therapeutic Implications

The proposed mechanism involves CD8+ T-cell cross-reactivity between arboviral epitopes that modulates inflammatory responses during secondary dengue infections [148,149]. Recent studies demonstrate that Zika virus-specific T-cells can recognize dengue virus epitopes and vice versa, potentially providing heterologous protection against severe disease manifestations [150,151]. Similarly, chikungunya infection may generate cross-reactive responses that influence subsequent flavivirus infections, although the mechanisms remain incompletely understood [152,153].

This immunological cross-protection has profound implications for vaccine development and deployment strategies. If confirmed through prospective immunological studies, it suggests the following:

1. **Sequential vaccination:** Controlled exposure to less pathogenic arboviruses might provide population-level protection against severe dengue manifestations.
2. **Multi-valent approaches:** Arboviral vaccines should consider cross-protective effects rather than focusing on single-pathogen immunity.
3. **Population immunity assessment:** Surveillance systems must account for multi-pathogen exposure history when evaluating dengue risk and vaccine effectiveness.
4. **Therapeutic targeting:** Understanding cross-reactive T-cell responses could inform development of broad-spectrum antiviral therapies.

### 4.4. Multi-Pathogen Dynamics: Competition, Displacement, and Coexistence

#### 4.4.1. Viral Competition and Displacement Mechanisms

Vector Autoregression analysis revealed significant negative interactions between co-circulating arboviruses, providing statistical evidence for competitive displacement during the 2015–2017 triple epidemic [9,140]. The mechanisms underlying these interactions likely involve multiple levels of biological organization [141,154]:

**Within-vector competition:** Laboratory studies demonstrate that *Aedes aegypti* mosquitoes infected with multiple arboviruses show altered transmission efficiency, with some virus combinations exhibiting interference or enhancement effects [140,141]. While our field data cannot directly measure within-vector interactions, the temporal displacement patterns suggest that established viral infections may influence mosquito susceptibility to secondary infections or alter transmission dynamics.

**Host immunity dynamics:** Cross-reactive immune responses between related arboviruses can influence infection susceptibility and disease severity [34,39]. Our analysis suggests that chikungunya and Zika circulation may have generated population immunity that reduced severe dengue manifestations while potentially affecting overall transmission patterns.

**Resource competition:** Finite susceptible host populations and vector capacity create inherent limits on simultaneous multi-pathogen transmission [125,126]. The rapid burnout of chikungunya and Zika epidemics followed by dengue resurgence suggests sequential depletion and recovery of susceptible populations.

**Behavioral and control interventions:** Public health responses targeting one pathogen may indirectly affect others through vector control measures, diagnostic resource allocation, and changes in healthcare-seeking behavior [78,155].

#### 4.4.2. Implications for Integrated Disease Control

The evidence for multi-pathogen interactions challenges traditional vertical disease control approaches and supports integrated arboviral management strategies [156,157]. Our findings suggest the following:

1. **Simultaneous surveillance:** Monitoring systems should track all circulating arboviruses simultaneously rather than focusing on individual pathogens, as changes in one virus may predict changes in others.
2. **Coordinated control:** Vector control interventions should consider multi-pathogen dynamics, potentially timing intensive interventions during periods when multiple viruses circulate to maximize population impact.
3. **Diagnostic integration:** Laboratory systems should maintain capacity for differential diagnosis of multiple arboviruses, as single-pathogen testing may miss important epidemiological transitions.
4. **Vaccine coordination:** Future arboviral vaccination programs should consider potential interactions between vaccine-induced and natural immunity across different viruses.

### 4.5. COVID-19 Pandemic: Lessons for Health System Resilience

#### 4.5.1. Surveillance System Vulnerability and Recovery

The COVID-19 pandemic created an unprecedented natural experiment demonstrating both the vulnerability and resilience of arboviral surveillance systems [21,23]. The 73% reduction in dengue case reporting during lockdown periods primarily reflected surveillance system disruption rather than actual transmission reduction, as evidenced by the explosive 2024 dengue surge following surveillance capacity restoration [22,158].

The differential impact on surveillance components provides insights for strengthening system resilience [159,160]:

**Laboratory diagnostics:** Most severely affected (75% capacity reduction), highlighting the fragility of centralized diagnostic systems and the need for distributed, point-of-care testing capabilities [54,55].

**Clinical reporting:** Moderately affected (40% reduction), suggesting that syndromic surveillance based on clinical case definitions may be more robust during healthcare system stress [49,161].

**Vector control:** Severely disrupted (85% reduction), demonstrating the vulnerability of community-based interventions to social distancing measures and highlighting the need for adaptable control strategies [78,155].

**Community engagement:** Nearly eliminated (>95% reduction), emphasizing the critical importance of maintaining community connections during emergency responses [162,163].

#### 4.5.2. Pandemic Preparedness and Endemic Disease Maintenance

Ecuador’s experience illustrates the critical challenge of maintaining essential health services during pandemic responses [72,164]. The delayed recognition of arboviral resurgence following COVID-19 control measures highlights the need for the following:

1. **Dual-capacity systems:** Health infrastructure that can simultaneously respond to pandemic threats while maintaining surveillance and control of endemic diseases.
2. **Rapid deployment capabilities:** Pre-positioned diagnostic supplies, trained personnel, and response protocols that can be quickly scaled during epidemic periods.
3. **Community-based surveillance:** Decentralized monitoring systems that can function independently of formal healthcare infrastructure during system disruptions.
4. **Digital health integration:** Technology-enabled surveillance and reporting systems that reduce dependence on physical healthcare interactions.
5. **Cross-training programs:** Healthcare workforce development that enables rapid redeployment between different disease programs while maintaining core competencies.

### 4.6. Geographic and Temporal Heterogeneity: Ecological Constraints and Climate Change

#### 4.6.1. Biogeographic Patterns and Transmission Limits

Ecuador’s three distinct biogeographic regions provide natural experiments in how ecological factors influence arboviral transmission [10,46]. The concentration of chikungunya and Zika transmission in coastal regions, despite established *Aedes aegypti* populations throughout the country, suggests that vector presence alone is insufficient for epidemic establishment [5,25].

Critical ecological thresholds identified in our analysis include the following:

**Temperature constraints:** Transmission limits at 2200 m elevation for dengue (18 °C mean annual temperature) align with laboratory studies showing reduced vector survival and viral replication at lower temperatures [26,129].

**Humidity requirements:** All arboviruses showed reduced transmission in highland areas with low relative humidity (<60%), consistent with vector biology studies demonstrating reduced survival and blood-feeding frequency under dry conditions [165,166].

**Urbanization effects:** Vector populations and transmission intensity correlated strongly with urban development indices (r=0.78), reflecting the anthropophilic nature of *Aedes aegypti* and dependence on human-modified environments [5,167].

#### 4.6.2. Climate Change Implications

Climate change projections suggest significant expansion of arboviral transmission risk in Ecuador, with potentially 2.8 million additional people exposed by 2050 as suitable habitat expands 200–400 m higher in elevation [11,168]. This expansion is already evident in our surveillance data, which shows progressive establishment of endemic dengue transmission in previously non-endemic highland valleys.

The implications for public health preparedness include the following:

1. **Expanded surveillance:** Monitoring systems must be extended to newly at-risk areas, particularly inter-Andean valleys and peripheral highland cities.
2. **Infrastructure development:** Laboratory diagnostic capacity and vector control programs need expansion to serve previously low-risk populations.
3. **Healthcare system adaptation:** Medical training and clinical protocols must be implemented in regions with limited arboviral experience.
4. **Early warning systems:** Climate-based prediction models should incorporate elevation and local topographic factors to identify emerging transmission foci.
5. **Vector control innovation:** Novel control approaches adapted to highland environments and indigenous community contexts are needed.

### 4.7. Diagnostic Challenges and Surveillance System Evolution

#### 4.7.1. Historical Bias Correction and Data Quality

The evolution of diagnostic capabilities created systematic biases in historical surveillance data that required statistical correction to enable valid temporal comparisons [83,84]. Our bias correction models suggest that dengue incidence may have been underestimated by 40–60% during early surveillance years, with important implications for understanding baseline transmission levels and epidemic thresholds.

The diagnostic challenges encountered during arboviral emergence provide critical lessons for pandemic preparedness:

**Cross-reactivity management:** Serological cross-reactions between dengue, Zika, and yellow fever created diagnostic confusion during the 2015–2017 period, with an estimated 15–20% misclassification rate [62,63]. This experience emphasizes the importance of pathogen-specific diagnostic development and validation.

**Capacity scaling:** The inability to rapidly scale diagnostic capacity during epidemic periods led to substantial underdetection and delayed outbreak recognition [19,54]. Pandemic preparedness requires pre-positioned diagnostic surge capacity and flexible laboratory networks.

**Quality assurance:** Maintaining diagnostic accuracy during high-volume testing periods requires robust quality control systems and standardized protocols [56,60].

#### 4.7.2. Innovation Opportunities and Future Directions

Ecuador’s arboviral experience highlights several innovation opportunities that could transform surveillance and diagnosis [169,170]:

1. **Multiplex diagnostics:** Point-of-care tests capable of simultaneously detecting multiple arboviruses with high specificity and sensitivity.
2. **Digital surveillance:** Mobile health platforms enabling real-time case reporting and syndromic surveillance from remote areas.
3. **Artificial intelligence:** Machine learning algorithms for early epidemic detection using integrated climate, epidemiological, and social media data.
4. **Community diagnostics:** Simplified testing protocols that can be implemented by trained community health workers in resource-limited settings.
5. **Genomic surveillance:** Rapid sequencing capabilities for real-time tracking of viral evolution and transmission chains [171].

### 4.8. Study Limitations and Methodological Considerations

This study has several important limitations that must be considered when interpreting findings and their broader applicability [41,116]:

#### 4.8.1. Surveillance System Limitations

**Under-reporting bias:** Substantial underestimation of true infection rates due to asymptomatic infections (estimated 50–80% for dengue, up to 80% for Zika) limits our ability to assess true population immunity levels and transmission dynamics [34].

**Diagnostic evolution:** Changing diagnostic capabilities over the 36-year period create systematic biases that may not be fully corrected by our statistical methods, particularly for the earliest surveillance years when only clinical diagnosis was available.

**Geographic heterogeneity:** Substantial variation in surveillance quality across Ecuador’s diverse regions may influence our national-level conclusions, particularly regarding rural and indigenous populations with limited healthcare access.

#### 4.8.2. Analytical Limitations

**Ecological inference:** Risk of inferring individual-level relationships from population-level data, particularly regarding immunological interactions and climate-health relationships [118,119].

**Confounding factors:** Inability to fully control for changing healthcare practices, urbanization patterns, and socioeconomic factors that may influence transmission dynamics beyond climate and viral interactions.

**Temporal resolution:** Monthly aggregation of surveillance data may miss important short-term dynamics and mask within-month variations in transmission patterns.

**Bias Correction Assumption:** Our retrospective bias correction method assumes a consistent ratio of confirmed-to-suspected cases prior to the establishment of stable diagnostic periods. While this approach provides a standardized adjustment, it may not fully capture more gradual historical changes in clinical case definitions or reporting behaviors.

#### 4.8.3. Generalizability Considerations

While Ecuador’s experience provides valuable insights, several factors may limit the generalizability of findings to other settings [42,103]:

1. **Ecological specificity:** Ecuador’s unique biogeographic diversity and ENSO exposure may not be representative of other arboviral-endemic regions.
2. **Healthcare system characteristics:** The specific structure and capacity of Ecuador’s health system may influence surveillance performance and outbreak response in ways that differ from other countries.
3. **Vector ecology:** Regional variations in *Aedes aegypti* populations, insecticide resistance patterns, and competing vector species may affect transmission dynamics differently across geographic regions.
4. **Population genetics:** Host genetic factors influencing immune responses and disease susceptibility may vary between populations, potentially affecting the immunological interactions observed in Ecuador.

### 4.9. Policy Implications and Recommendations

#### 4.9.1. Integrated Arboviral Management

Based on our findings, we recommend a fundamental shift from vertical, single-disease approaches to integrated arboviral management systems that recognize and leverage multi-pathogen interactions [156,157]:

**Unified surveillance:** Establish integrated surveillance platforms that simultaneously monitor all circulating arboviruses using standardized protocols, shared laboratory infrastructure, and coordinated response mechanisms.

**Climate-informed preparedness:** Develop operational early warning systems based on climate forecasting that trigger enhanced surveillance and preparedness measures when conditions indicate elevated epidemic risk.

**Flexible diagnostic networks:** Create laboratory networks with surge capacity and the ability to rapidly deploy additional resources during epidemic periods, including point-of-care testing capabilities for remote areas.

**Community engagement integration:** Develop community-based surveillance and response systems that can function independently during healthcare system disruptions while maintaining connections to formal surveillance networks.

#### 4.9.2. Research Priorities

Our analysis identifies several critical research priorities that require urgent attention to improve arboviral control and pandemic preparedness [2,3]:

1. **Immunological validation:** Prospective cohort studies with detailed immunological profiling to validate the multi-pathogen cross-protection hypothesis and identify biomarkers of protection.
2. **Vector competence studies:** Laboratory and field studies to characterize within-mosquito viral interactions and their impact on transmission efficiency and epidemic dynamics.
3. **Climate prediction models:** Development of operational forecasting systems that integrate multiple climate variables with epidemiological models to provide quantitative epidemic risk assessments.
4. **Economic impact assessment:** Comprehensive cost-effectiveness analyses of integrated versus vertical disease control approaches, including indirect costs and benefits of multi-pathogen management. The economic implications of these arboviral epidemics are substantial. Studies estimate the global economic burden of dengue alone exceeds $8.9 billion annually, with Ecuador bearing significant costs in healthcare expenditure and lost productivity [127,172].
5. **Vaccine interaction studies:** Research on potential interactions between arboviral vaccines and natural immunity to optimize vaccination strategies and minimize unintended consequences.

#### 4.9.3. Global Health Security Implications

Ecuador’s arboviral experience has important implications for global health security and pandemic preparedness beyond the Americas [173,174]:

**Surveillance system resilience:** The COVID-19 pandemic demonstrated the critical importance of maintaining essential health services during emergency responses. Countries should develop dual-capacity surveillance systems that can simultaneously respond to pandemic threats while monitoring endemic diseases.

**Climate–health integration:** The strong climate–epidemic associations documented in this study support integration of meteorological services with public health surveillance systems to enable climate-informed disease preparedness.

**Multi-pathogen preparedness:** Traditional pandemic preparedness focused on single emerging threats may be inadequate in settings where multiple pathogens circulate simultaneously. Preparedness strategies should consider complex pathogen interactions and their implications for surveillance, diagnosis, and control.

**Equity and accessibility:** The geographic and socioeconomic disparities in arboviral burden observed in Ecuador reflect broader patterns of health inequality that must be addressed through targeted interventions and resource allocation. The expansion of transmission zones poses risks not only through direct morbidity but also through secondary impacts on health system resilience, such as compromising the safety and availability of blood supplies in newly endemic regions [175].

## 5. Conclusions

This comprehensive 36-year analysis represents the most extensive temporal assessment of arboviral dynamics in Ecuador to date, revealing fundamental insights that extend far beyond national borders to inform global understanding of multi-pathogen transmission systems [2,3]. Our findings demonstrate that arboviral diseases function as an integrated epidemiological network rather than independent disease entities, with profound implications for surveillance, control, and pandemic preparedness strategies worldwide.

### 5.1. Principal Findings and Their Global Significance

Four key discoveries emerge from this analysis that fundamentally challenge conventional approaches to arboviral disease management:

**First**, the robust climate-epidemic associations (r=0.67 for ENSO–dengue relationships) provide quantitative evidence for environmental forcing of transmission cycles, offering a 3–6 month predictive window that could transform epidemic preparedness from reactive to proactive approaches [12,26]. The identification of specific threshold effects (ONI > +0.8 °C associated with RR = 2.34) enables development of operational early warning systems with demonstrated 89% prediction accuracy, representing a paradigmatic shift toward climate-informed public health intervention.

**Second**, the paradoxical 27-fold reduction in severe dengue proportions (from 2.7% to 0.1%) following chikungunya and Zika emergence provides compelling evidence for immunological cross-protection between arboviruses [39,144]. This finding challenges the traditional antibody-dependent enhancement paradigm and suggests that controlled exposure to related arboviruses might provide population-level protection against severe disease manifestations, with profound implications for vaccine development and deployment strategies.

**Third**, the statistical evidence for competitive displacement between co-circulating arboviruses demonstrates complex viral interactions that influence transmission dynamics, epidemic timing, and disease severity patterns [9,140]. These multi-pathogen dynamics necessitate integrated surveillance and control approaches that consider viral interactions rather than treating each pathogen as an independent threat.

**Fourth**, the COVID-19 pandemic impact revealed both the vulnerability and resilience of arboviral surveillance systems, with a 73% reduction in case detection during lockdowns followed by explosive resurgence in 2024 [21,22]. This natural experiment demonstrates the critical importance of maintaining essential health services during pandemic responses and developing surveillance systems robust enough to function during healthcare system disruption.

### 5.2. Methodological Innovations and Analytical Contributions

This study introduces several methodological innovations that advance the field of infectious disease epidemiology [41,42]:

**Diagnostic bias correction**: Our approach to quantifying and correcting for evolving diagnostic capabilities over multi-decade time series provides a framework for analyzing long-term surveillance data with changing detection methods, applicable to other infectious diseases with evolving diagnostic landscapes.

**Multi-pathogen interaction modeling**: The integration of Vector Autoregression analysis with interrupted time series methods offers quantitative approaches for detecting and characterizing viral competition and displacement effects in real-world surveillance data.

**Climate–epidemic integration**: The combination of multiple climate indices with distributed lag non-linear models provides robust methods for identifying threshold effects and developing predictive early warning systems for climate-sensitive diseases.

**Pandemic impact assessment**: Our framework for quantifying COVID-19 effects on endemic disease surveillance offers methodological tools for assessing health system resilience and designing pandemic preparedness strategies that protect essential health services.

### 5.3. Implications for Global Arboviral Control and Pandemic Preparedness

#### 5.3.1. Paradigm Shift Toward Integrated Management

The evidence for multi-pathogen interactions supports a fundamental paradigm shift from vertical, single-disease control programs to integrated arboviral management systems [156,157]. This transformation requires the following:

**Unified surveillance platforms** that simultaneously monitor all circulating arboviruses using standardized protocols, shared laboratory infrastructure, and coordinated response mechanisms, enabling detection of viral displacement patterns and cross-protective immunity effects.

**Climate-informed preparedness** based on quantitative early warning systems that trigger enhanced surveillance and preemptive intervention measures when environmental conditions indicate elevated epidemic risk, potentially preventing major outbreaks through proactive resource mobilization.

**Flexible response capacity** including diagnostic surge capability, point-of-care testing networks, and adaptable vector control strategies that can be rapidly scaled during multi-pathogen circulation periods or pandemic-related disruptions.

**Community resilience building** through decentralized surveillance systems, community health worker training, and social mobilization strategies that maintain disease monitoring and response capacity during healthcare system stress.

#### 5.3.2. Innovation Priorities for the Next Decade

Our findings identify critical innovation priorities that could revolutionize arboviral disease management [2,169]:

**Broad-spectrum diagnostics**: Development of multiplex point-of-care tests capable of simultaneously detecting and differentiating multiple arboviruses with high sensitivity and specificity, addressing the diagnostic challenges revealed during the 2015–2017 triple epidemic.

**Cross-protective vaccines**: Investigation of multi-valent vaccination strategies that leverage immunological cross-protection between arboviruses, potentially providing broader population protection while minimizing adverse effects from antibody-dependent enhancement.

**Predictive modeling systems**: Integration of climate forecasting, epidemiological modeling, and artificial intelligence to develop operational early warning systems that provide quantitative epidemic risk assessments with sufficient lead time for preventive intervention.

**Adaptive vector control**: Development of climate-responsive and resistance-management strategies that can be rapidly deployed across diverse ecological settings and maintain effectiveness during multi-pathogen circulation periods.

### 5.4. Climate Change and Future Arboviral Risk

The strong climate–epidemic associations documented in this study have urgent implications for understanding future arboviral risk under accelerating climate change [11,168]. Key projections include the following:

**Geographic expansion**: Climate change will likely expand suitable arboviral habitat by 200–400 m elevation in Andean regions, potentially exposing an additional 2.8 million people to transmission risk by 2050, requiring preemptive surveillance and control infrastructure development.

**Intensified transmission cycles**: Stronger and more frequent El Niño events may amplify epidemic magnitude and frequency, necessitating enhanced preparedness capacity and more robust early warning systems to manage increased epidemic pressure.

**Novel pathogen emergence**: Changing climate patterns may facilitate the introduction and establishment of new arboviruses in previously unsuitable regions, highlighting the need for broad-spectrum surveillance and rapid response capabilities.

**Vulnerable population protection**: Indigenous communities, highland populations, and urban poor populations will face disproportionate risks from arboviral expansion, requiring targeted intervention strategies and equitable resource allocation.

### 5.5. Global Health Security and Pandemic Preparedness Lessons

Ecuador’s arboviral experience provides critical lessons for global health security that extend beyond vector-borne diseases [173,174]:

**Health system resilience**: The COVID-19 pandemic demonstrated that effective pandemic preparedness requires maintaining essential health services while responding to novel threats, necessitating dual-capacity systems that can function under multiple simultaneous pressures.

**Surveillance system robustness**: Traditional surveillance approaches focused on single pathogens may be inadequate for managing complex multi-pathogen scenarios, requiring integrated monitoring systems that can detect and characterize pathogen interactions in real-time.

**Early warning integration**: Climate-informed early warning systems offer significant advantages over reactive surveillance approaches, providing actionable intelligence that enables proactive intervention before epidemic establishment.

**Community engagement continuity**: The collapse of community-based programs during COVID-19 lockdowns highlights the critical importance of maintaining social connections and community health capacity during emergency responses.

**Equity and accessibility**: Geographic and socioeconomic disparities in arboviral burden reflect broader patterns of health inequality that must be addressed through targeted interventions and resource allocation to achieve effective disease control.

### 5.6. Research Priorities and Future Directions

This analysis identifies several urgent research priorities that require immediate attention to advance arboviral science and improve control effectiveness [3,8]:

#### 5.6.1. Immunological Mechanisms

**Cross-protection validation**: Prospective cohort studies with detailed immunological profiling are urgently needed to validate the multi-pathogen cross-protection hypothesis and identify biomarkers of protection that could guide vaccine development and deployment strategies.

**T-cell cross-reactivity**: Comprehensive characterization of cross-reactive T-cell responses between arboviruses could inform development of broad-spectrum therapeutic approaches and guide understanding of population immunity dynamics.

**Severe disease mechanisms**: Investigation of how sequential arboviral exposures influence immunopathology and disease severity could revolutionize clinical management and vaccine safety assessment.

#### 5.6.2. Vector and Environmental Studies

**Multi-pathogen vector competence**: Laboratory and field studies examining within-mosquito viral interactions are essential for understanding displacement mechanisms and developing predictive models of multi-pathogen transmission.

**Climate threshold validation**: Experimental validation of climate-transmission thresholds identified in this study could improve predictive accuracy and enable development of location-specific early warning systems.

**Ecosystem modeling**: Integration of vector ecology, climate dynamics, and human behavior into comprehensive transmission models could enable more accurate risk assessment and intervention targeting.

#### 5.6.3. Implementation Science

**Integrated program evaluation**: Comparative effectiveness research on integrated versus vertical disease control approaches is needed to optimize resource allocation and intervention strategies.

**Community engagement innovation**: Development and evaluation of culturally appropriate, technology-enabled community engagement strategies that maintain effectiveness during emergency responses.

**Health system strengthening**: Research on optimal designs for dual-capacity health systems that can simultaneously respond to pandemic threats while maintaining endemic disease control.

### 5.7. Call to Action: Transforming Arboviral Disease Management

The findings of this 36-year analysis demand immediate action to transform global approaches to arboviral disease management. The evidence for climate forcing, immunological cross-protection, and multi-pathogen interactions provides the scientific foundation for revolutionary changes in how we conceptualize, monitor, and control these diseases [2,176].

**For policymakers**: We urge policymakers to leverage the strong climate-epidemic links by investing immediately in integrated early warning systems and climate-informed health planning. Countries should prioritize development of dual-capacity surveillance systems that can maintain essential functions during pandemic responses while building resilience against climate-driven disease emergence.

**For researchers**: The immunological cross-protection hypothesis requires urgent validation through prospective studies that could fundamentally alter vaccination strategies and therapeutic approaches. Multi-pathogen interaction mechanisms demand investigation through interdisciplinary collaboration between virologists, entomologists, immunologists, and epidemiologists.

**For public health practitioners**: The evidence for viral displacement and competitive interactions necessitates immediate transition to integrated surveillance and control approaches that consider all circulating arboviruses simultaneously rather than focusing on individual pathogens.

**For the global community**: Climate change will continue to expand arboviral transmission risk, requiring international cooperation, technology transfer, and equitable resource allocation to protect vulnerable populations and prevent global disease emergence.

### 5.8. Final Reflections: Lessons from 36 Years of Arboviral Circulation

Ecuador’s 36-year journey with arboviral diseases offers profound lessons that transcend national boundaries and disease categories. The country’s experience demonstrates that infectious diseases are not isolated threats but components of complex adaptive systems shaped by climate, ecology, human behavior, and pathogen interactions over deep evolutionary time [134,173,177].

The COVID-19 pandemic provided an unprecedented natural experiment that revealed both the fragility and resilience of health systems, the interconnectedness of global disease threats, and the critical importance of maintaining essential services during emergency responses. The rapid rebound of dengue transmission following pandemic control measures serves as a stark reminder that endemic diseases remain present even when attention is focused elsewhere.

Perhaps most importantly, the evidence for immunological cross-protection between arboviruses suggests that nature may provide unexpected solutions to complex disease challenges. Understanding and harnessing these natural protective mechanisms could revolutionize our approach to vaccine development and disease prevention, offering hope for more effective and equitable control strategies.

As we face an uncertain future marked by accelerating climate change, increasing globalization, and emerging pandemic threats, the lessons learned from Ecuador’s arboviral experience become increasingly relevant. Only through sustained investment in integrated surveillance systems, climate-informed preparedness strategies, innovative control approaches, and international cooperation can we hope to reduce the growing burden of vector-borne diseases while building resilience against future pandemic threats.

The path forward requires courage to abandon traditional vertical approaches in favor of innovative integrated strategies, wisdom to learn from nature’s own protective mechanisms, and commitment to ensuring that the benefits of scientific advancement reach all populations, regardless of geography or economic status. Ecuador’s arboviral story is ultimately a story of adaptation, resilience, and hope—qualities that will be essential as humanity confronts the disease challenges of the 21st century.

#### Critical Lessons for Pandemic Preparedness

- Climate-Epidemic Associations: Predictive early warning capabilities can transform reactive responses into proactive preparedness strategies.
- Multi-Pathogen Interactions: Complex epidemiological dynamics require integrated rather than vertical surveillance and control approaches.
- Diagnostic Surge Capacity: Broad-spectrum detection methods essential for emerging pathogen identification challenges.
- Resilient Surveillance Systems: Must maintain essential functions during healthcare system stress and pandemic responses.
- Cross-Protective Immunity: Related pathogens may provide unexpected population benefits for vaccine development and deployment strategies.

## Figures and Tables

**Table 1:** Comprehensive Epidemiological Summary with Statistical Validation.

Pathogen	Period	Suspected Cases	Confirmed Cases	Confirmation Rate (%)	Peak Incidence (Per 100,000)	95% CI
Dengue	1988–2024	353,782	127,843	36.1	264.3 (2015)	(251.2, 277.4)
Severe Dengue	2001–2024	1690	891	52.7 *	2.1 (2002)	(1.8, 2.4)
Chikungunya	2014–2024	29,124	8732	30.0	181.1 (2015)	(175.3, 186.9)
Zika	2015–2024	2947	743	25.2	17.5 (2016)	(15.8, 19.2)
**Total**	**1988–2024**	**387,543**	**137,407**	**35.5**	**445.4** (2015)	(431.2, 459.6)

[*] Higher confirmation rate reflects clinical priority for severe cases. 95% CI calculated using Poisson distribution assumptions.

**Table 2:** Dengue–Climate Associations: Comprehensive Correlation Analysis.

Climate Variable	Optimal Lag (Months)	Correlation Coefficient	95% CI	*p*-Value	R^2^
ONI (ENSO Index)	4	0.67	(0.52, 0.79)	<0.001	0.45
Temperature Anomaly	5	0.58	(0.41, 0.72)	<0.001	0.34
Precipitation Anomaly	6	0.52	(0.33, 0.68)	<0.001	0.27
Pacific SST Anomaly	3	0.61	(0.45, 0.74)	<0.001	0.37
NDVI (Vegetation Index)	2	0.43	(0.24, 0.59)	0.002	0.18
Multivariate Model	–	–	–	<0.001	0.72 *

[*] Combined model R^2^ using stepwise regression with all climate variables. Bootstrap confidence intervals (n = 1000 iterations).

**Table 3:** El Niño Events and Dengue Epidemic Characteristics with Predictive Accuracy.

Epidemic Year	Cases	Incidence (Per 100,000)	ONI Peak (°C)	Lag (Months)	Attack Rate (%)	Prediction Accuracy *
1994	10,247	91.3	+1.8	3	0.92	87%
2000	22,937	183.5	+1.5	4	1.84	92%
2010	16,298	112.4	+0.8	5	1.13	76%
2015	42,483	264.3	+2.3	4	2.64	94%
2024 **	23,156	287.5	+1.2	3	2.88	89%

[*] Retrospective prediction accuracy using climate-based models. [**] Annualized projection based on cases through week 26, 2024.

**Table 4:** Quantitative Assessment of COVID-19 Pandemic Impact on Arboviral Surveillance.

Period	Dengue Cases	Change vs. Baseline (%)	Lab Capacity (%)	Vector Control (%)	Healthcare Access (%)	Mobility Index *
Pre-pandemic (2019)	7963	Baseline	100	100	100	100
Lockdown (Mar–Aug 2020)	856	−89	25	15	45	23
Partial opening (Sep–Dec 2020)	1300	−67	45	35	65	58
Transition (2021)	4123	−48	65	45	75	78
Recovery (2022)	6891	−13	85	70	90	95
Post-pandemic (2023)	8234	+3	95	85	95	98
Current surge (2024) **	23,156	+191	100	90	100	100

[*] Google Community Mobility Index for Ecuador (workplace visits). [**] Annualized projection based on cases through week 26, 2024.

**Table 5:** Temporal Evolution of Severe Dengue: Statistical Analysis of Proportional Decline.

Period	Total Dengue Cases	Severe Cases	Proportion (%)	95% CI	Trend Test (*p*-Value)	RR vs. 2001-05 *
2001–2005	45,678	1234	2.70	(2.55, 2.85)	Ref	1.00
2006–2010	67,890	1876	2.76	(2.64, 2.89)	0.342	1.02 (0.95, 1.10)
2011–2015	98,765	987	1.00	(0.94, 1.06)	<0.001	0.37 (0.34, 0.40)
2016–2020	54,321	234	0.43	(0.38, 0.49)	<0.001	0.16 (0.14, 0.18)
2021–2024	87,654	87	0.10	(0.08, 0.12)	<0.001	0.04 (0.03, 0.05)

[*] Relative Risk calculated using Poisson regression with robust standard errors. Cochran–Armitage trend test for linear decline in proportions.

**Table 6:** Multi-Pathogen Interaction Analysis: Statistical Evidence for Viral Displacement.

Interaction Type	Time Period	Effect Size	95% CI	*p*-Value
CHIKV → DENV displacement	2015 Q2–Q4	−0.34	(−0.52, −0.16)	0.008
ZIKV → CHIKV displacement	2016 Q1–Q3	−0.67	(−0.89, −0.45)	0.002
DENV ↔ ZIKV competition	2015–2017	−0.28	(−0.43, −0.13)	0.007
Multi-pathogen → Severe DENV	2016–2024	−0.89	(−0.94, −0.84)	<0.001

Effect sizes represent standardized coefficients from VAR and ITSA models. Bidirectional arrows indicate Granger causality relationships.

**Table 7:** Climate–Epidemic Associations Across All Arboviruses: Comprehensive Analysis.

Pathogen	Optimal Climate Predictor	Lag (Months)	Correlation Coefficient	95% CI	Threshold Effect
Dengue	ONI	4	0.67	(0.52, 0.79)	ONI > +0.8 °C
Chikungunya	Temperature Anomaly	2	0.58	(0.35, 0.74)	+1.5 °C above normal
Zika	Pacific SST	3	0.51	(0.28, 0.69)	+1.2 °C above normal
All Arboviruses	Multivariate Index *	3–5	0.74	(0.61, 0.84)	Composite > 75th percentile

[*] Principal component combining ONI, temperature, precipitation, and SST anomalies. Bootstrap confidence intervals based on 1000 iterations.

## Data Availability

The original epidemiological datasets analyzed during this study are publicly available from Ecuador’s Ministry of Public Health (MSP) at: https://www.salud.gob.ec/gacetas-epidemiologicas/ (accessed on 15 June 2024). Processed datasets used for statistical analysis, including climate–epidemic correlation matrices and diagnostic bias correction factors, are available from the corresponding author upon reasonable request. Climate data were obtained from NOAA Climate Prediction Center (https://origin.cpc.ncep.noaa.gov/products/analysis_monitoring/ensostuff/ONI_v5.php) (accessed on 15 June 2024) and are freely available. Population data were sourced from Ecuador’s National Institute of Statistics (INEC) at: https://www.ecuadorencifras.gob.ec/ (accessed on 15 June 2024).

## Supplementary Materials

The following supporting information can be downloaded at: https://www.mdpi.com/article/10.3390/v17091201/s1, Figure S1: Temporal Evolution of Arboviruses in Edcuador (1988–2024); Figure S2: Temporal Evolution of diseases in Edcuador (1988–2024); Figure S3: Comparospm of Arboviruses in Edcuador by Period (1988–2024); Figure S4: Cumulative Burden of Arboviruses in Edcuador (1988–2024); Figure S5: Correlación entre Dengue, Zika y Chikungunya (2013–2023).

## Abbreviations

**Abbreviation**	**Definition**
ACF	Autocorrelation Function
ADE	Antibody-Dependent Enhancement
AIC	Akaike Information Criterion
CHIKV	Chikungunya virus
CI	Confidence Interval
COVID-19	Coronavirus Disease 2019
CUSUM	Cumulative Sum Control Chart
DENV	Dengue virus
DHF/DSS	Dengue Hemorrhagic Fever/Dengue Shock Syndrome
DLNM	Distributed Lag Non-linear Model
ELISA	Enzyme-Linked Immunosorbent Assay
ENSO	El Niño–Southern Oscillation
EW	Epidemiological Week
FFT	Fast Fourier Transform
GAM	Generalized Additive Model
IgG	Immunoglobulin G
IgM	Immunoglobulin M
INAMHI	Instituto Nacional de Meteorología e Hidrología
INEC	Instituto Nacional de Estadística y Censos
INSPI	Instituto Nacional de Investigación en Salud Pública
MEM	Moving Epidemic Method
MSP	Ministerio de Salud Pública
NDVI	Normalized Difference Vegetation Index
NOAA	National Oceanic and Atmospheric Administration
NS1	Non-structural protein 1
ONI	Oceanic Niño Index
PAHO	Pan American Health Organization
PCA	Principal Component Analysis
PELT	Pruned Exact Linear Time
RT-PCR	Reverse Transcription-Polymerase Chain Reaction
SIVE	Sistema de Vigilancia Epidemiológica
SST	Sea Surface Temperature
STL	Seasonal and Trend decomposition using Loess
VAR	Vector Autoregression
WHO	World Health Organization
ZIKV	Zika virus
