# Supplementary material for: Time Series Analysis of Dengue, Zika, and Chikungunya in Ecuador: Emergence Patterns, Epidemiological Interactions, and Climate-Driven Dynamics (1988–2024)
Source: Viruses. 2025 Aug 31;17(9):1201. doi: 10.3390/v17091201 (PMC12474222; doi:10.3390/v17091201)
Supplement: Supplementary file 1 [file viruses-17-01201-s001.zip › viruses-3765486-supplementary.pdf]

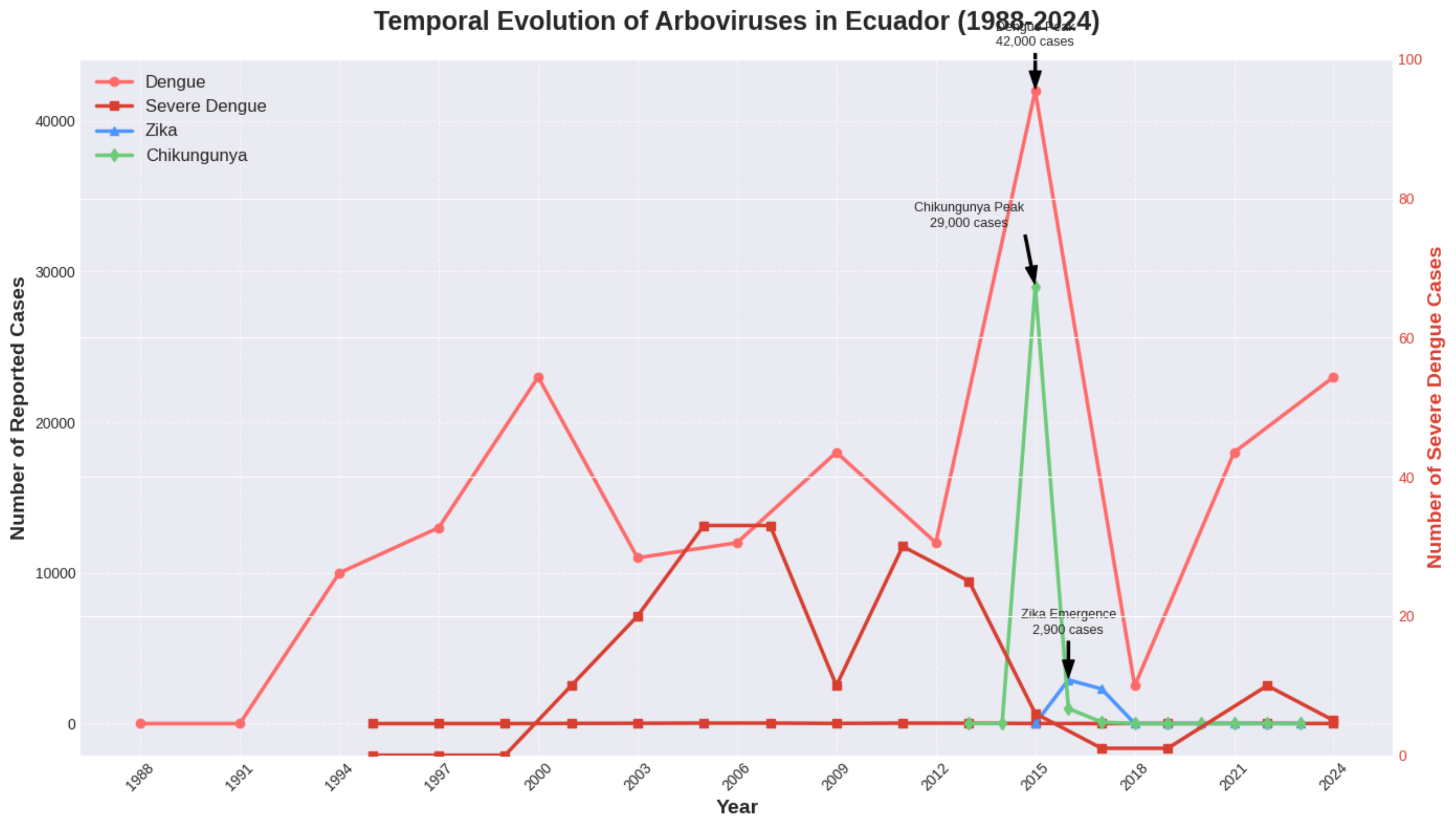

Temporal Evolution of Arboviruses in Ecuador (1988-2024)
Dengue
Severe Dengue
Zika
Chikungunya
Dengue Peak
42,000 cases
Chikungunya Peak
29,000 cases
Zika Emergence
2,900 cases
Number of Reported Cases
Number of Severe Dengue Cases
Year
0
10000
20000
30000
40000
0
20
40
60
80
100
1988
1991
1994
1997
2000
2003
2006
2009
2012
2015
2018
2021
2024

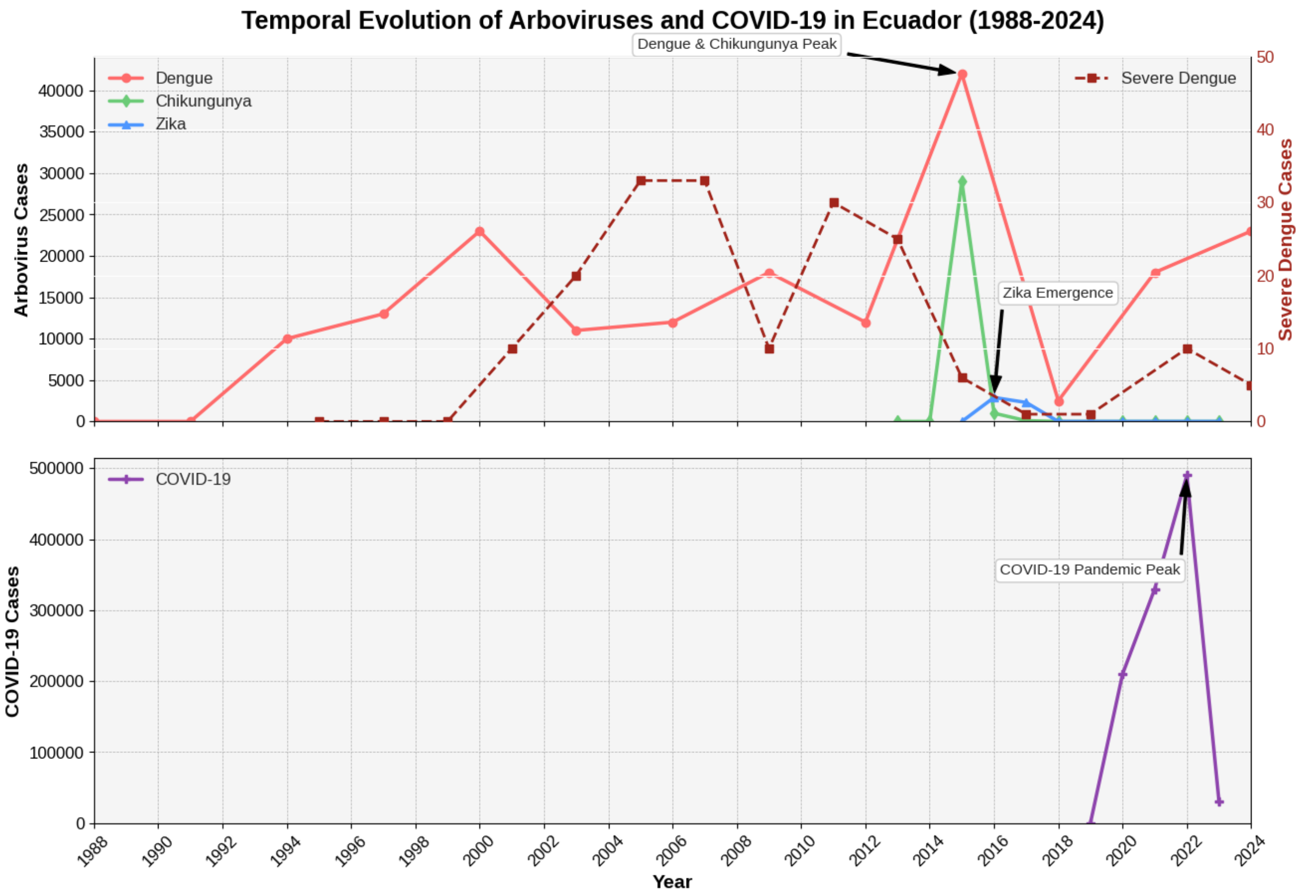

Temporal Evolution of Arboviruses and COVID-19 in Ecuador (1988-2024)
Dengue & Chikungunya Peak
Dengue
Chikungunya
Zika
Severe Dengue
Zika Emergence
Arbovirus Cases
Severe Dengue Cases
COVID-19
COVID-19 Pandemic Peak
COVID-19 Cases
Year

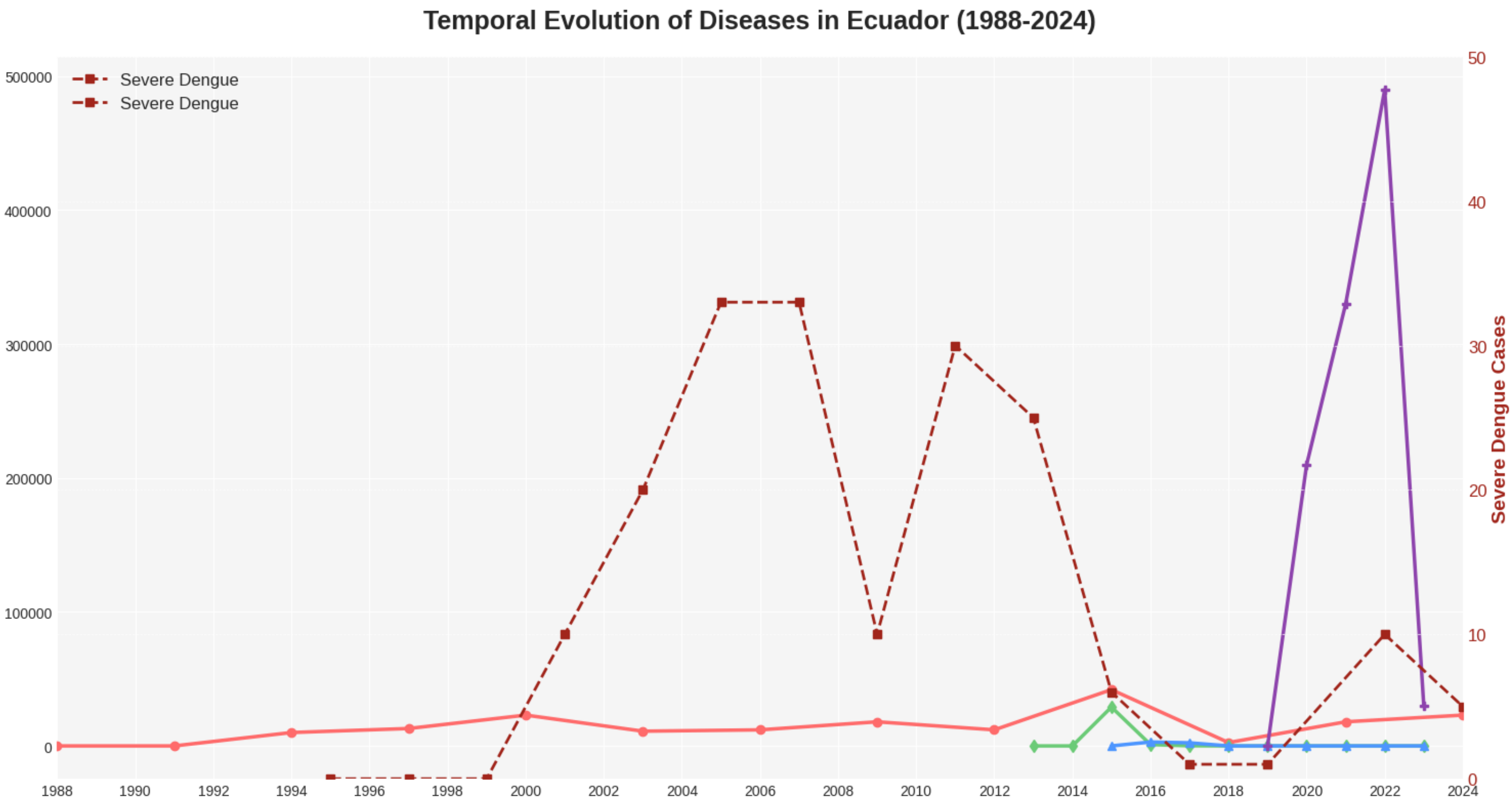

Temporal Evolution of Diseases in Ecuador (1988-2024)
Severe Dengue
Severe Dengue
500000
400000
300000
200000
100000
0
1988
1990
1992
1994
1996
1998
2000
2002
2004
2006
2008
2010
2012
2014
2016
2018
2020
2022
2024
50
40
30
20
10
0
Severe Dengue Cases

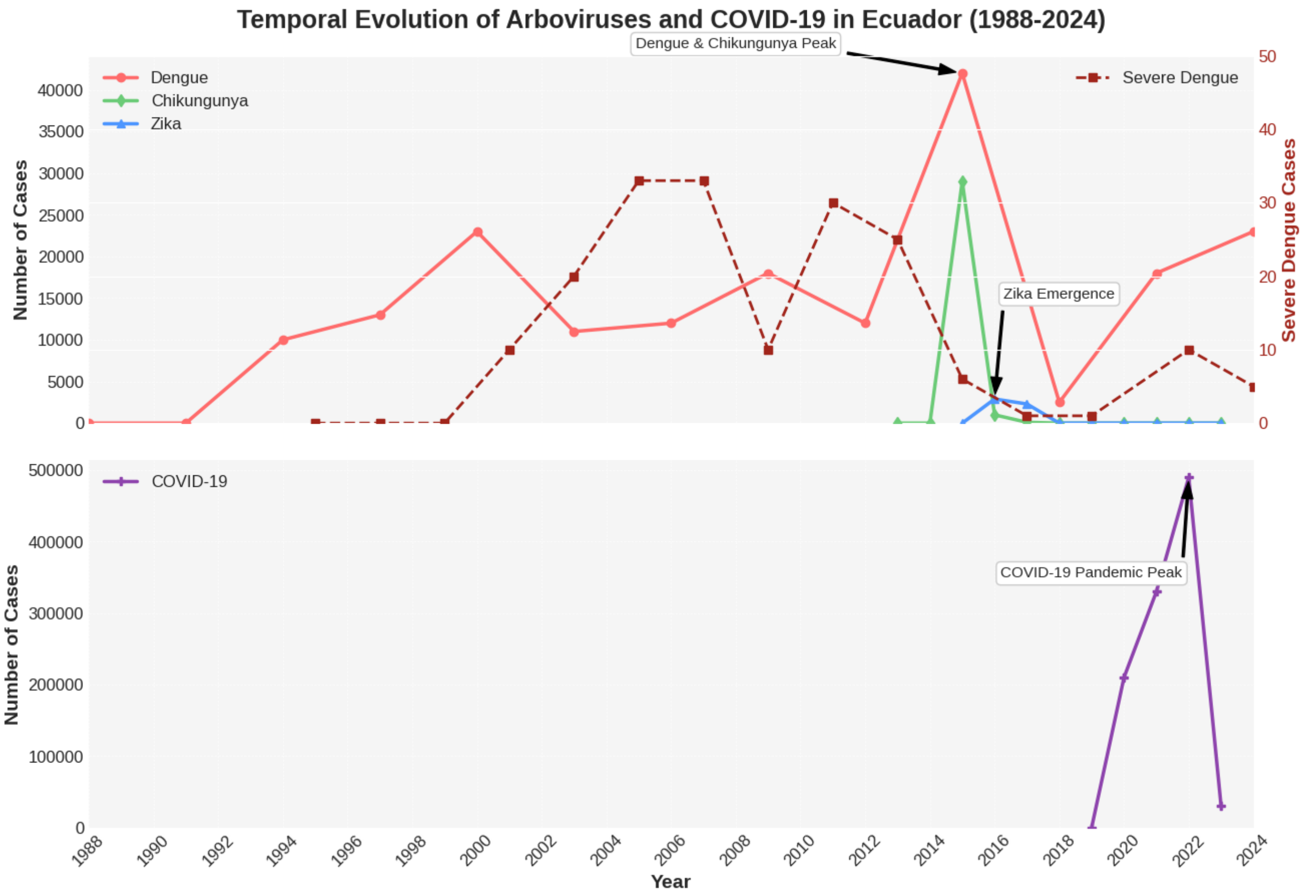
Temporal Evolution of Arboviruses and COVID-19 in Ecuador (1988-2024)
Dengue & Chikungunya Peak
Dengue
Chikungunya
Zika
Severe Dengue
Zika Emergence
Number of Cases
Severe Dengue Cases
COVID-19
COVID-19 Pandemic Peak
Year

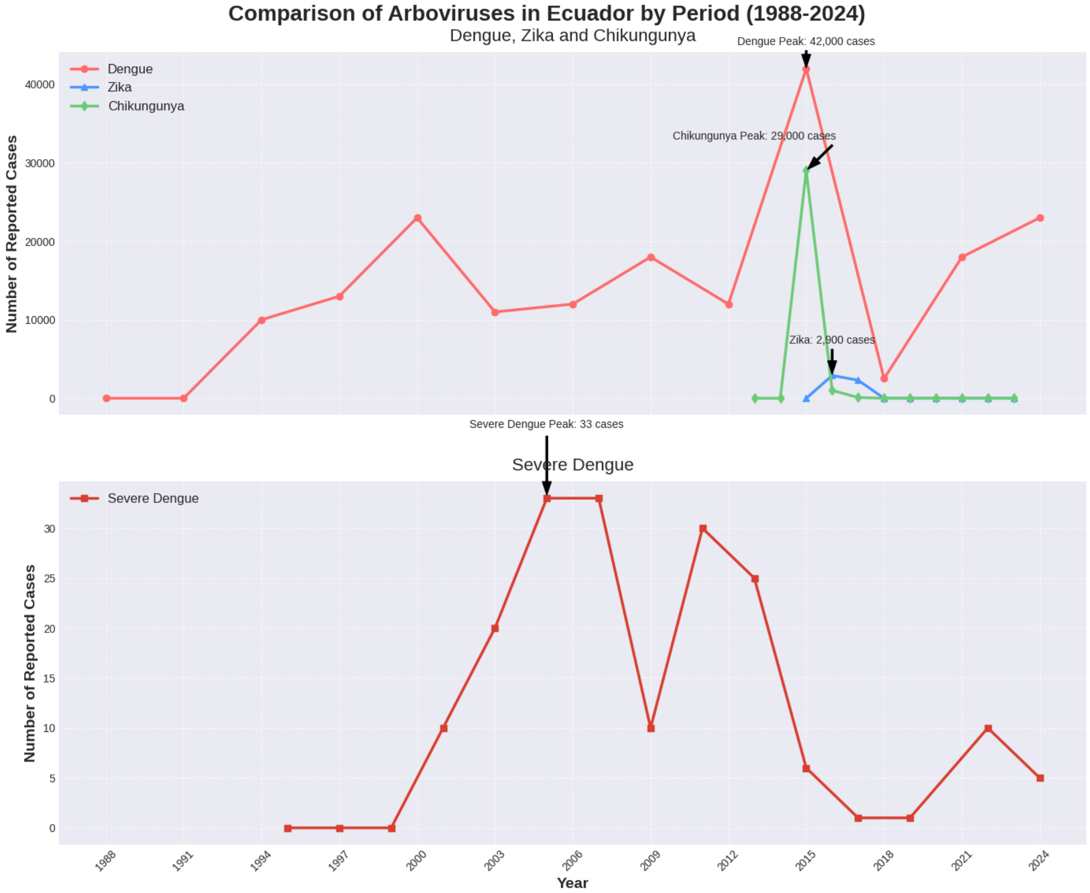

Comparison of Arboviruses in Ecuador by Period (1988-2024)
Dengue, Zika and Chikungunya
Dengue Peak: 42,000 cases
Dengue
Zika
Chikungunya
Chikungunya Peak: 29,000 cases
Zika: 2,900 cases
Number of Reported Cases
Severe Dengue Peak: 33 cases
Severe Dengue
Severe Dengue
Number of Reported Cases
Year

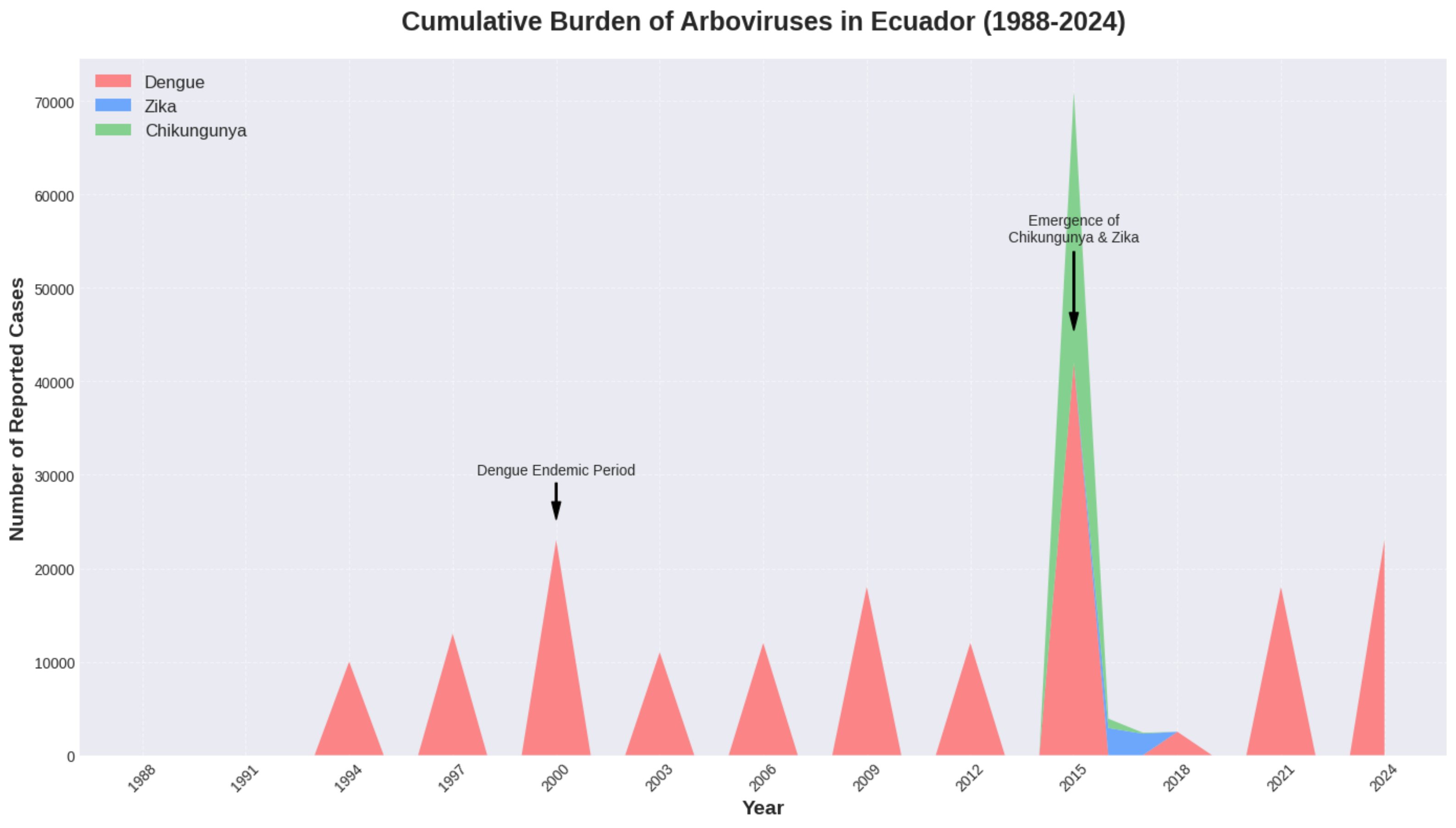
Cumulative Burden of Arboviruses in Ecuador (1988-2024)
Dengue
Zika
Chikungunya
Emergence of
Chikungunya & Zika
Dengue Endemic Period
Number of Reported Cases
Year
0
10000
20000
30000
40000
50000
60000
70000
1988
1991
1994
1997
2000
2003
2006
2009
2012
2015
2018
2021
2024

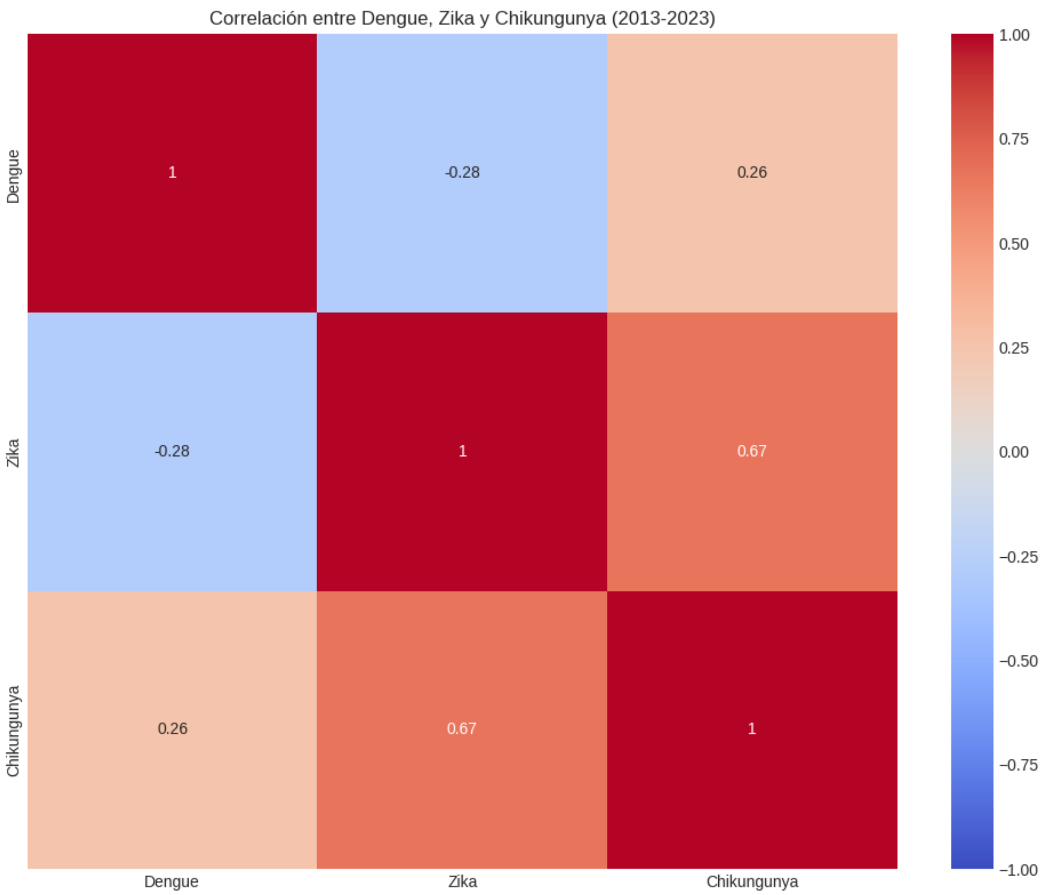
Correlación entre Dengue, Zika y Chikungunya (2013-2023)
Dengue
Zika
Chikungunya
1
-0.28
0.26
-0.28
1
0.67
0.26
0.67
1
Dengue
Zika
Chikungunya
1.00
0.75
0.50
0.25
0.00
−0.25
−0.50
−0.75
−1.00
